# Quantum-mechanical effects in photoluminescence from thin crystalline gold films

**DOI:** 10.1038/s41377-024-01408-2

**Published:** 2024-04-19

**Authors:** Alan R. Bowman, Alvaro Rodríguez Echarri, Fatemeh Kiani, Fadil Iyikanat, Ted V. Tsoulos, Joel D. Cox, Ravishankar Sundararaman, F. Javier García de Abajo, Giulia Tagliabue

**Affiliations:** 1 Laboratory of Nanoscience for Energy Technologies (LNET), STI, École Polytechnique Fédérale de Lausanne (EPFL), Lausanne, Switzerland; 2 ICFO–Institut de Ciencies Fotoniques, The Barcelona Institute of Science and Technology, Castelldefels (Barcelona), Spain; 3 MBI–Max-Born-Institut, Berlin, Germany; 4 POLIMA–Center for Polariton-driven Light–Matter Interactions, University of Southern Denmark, Odense M, Denmark; 5 Danish Institute for Advanced Study, University of Southern Denmark, Odense M, Denmark; 6 Department of Materials Science & Engineering, Rensselaer Polytechnic Institute, Troy, NY USA; 7 Department of Physics, Applied Physics, and Astronomy, Rensselaer Polytechnic Institute, Troy, NY USA; 8 ICREA–Institució Catalana de Recerca i Estudis Avançats, Barcelona, Spain

**Keywords:** Nanophotonics and plasmonics, Fluorescence spectroscopy, Nanophotonics and plasmonics

## Abstract

Luminescence constitutes a unique source of insight into hot carrier processes in metals, including those in plasmonic nanostructures used for sensing and energy applications. However, being weak in nature, metal luminescence remains poorly understood, its microscopic origin strongly debated, and its potential for unraveling nanoscale carrier dynamics largely unexploited. Here, we reveal quantum-mechanical effects in the luminescence emanating from thin monocrystalline gold flakes. Specifically, we present experimental evidence, supported by first-principles simulations, to demonstrate its photoluminescence origin (i.e., radiative emission from electron/hole recombination) when exciting in the interband regime. Our model allows us to identify changes to the measured gold luminescence due to quantum-mechanical effects as the gold film thickness is reduced. Excitingly, such effects are observable in the luminescence signal from flakes up to 40 nm in thickness, associated with the out-of-plane discreteness of the electronic band structure near the Fermi level. We qualitatively reproduce the observations with first-principles modeling, thus establishing a unified description of luminescence in gold monocrystalline flakes and enabling its widespread application as a probe of carrier dynamics and light-matter interactions in this material. Our study paves the way for future explorations of hot carriers and charge-transfer dynamics in a multitude of material systems.

## Abstract (web summary)

This work studies luminescence from monocrystalline gold flakes down to 13 nm thick. It reveals this signal is photoluminescence, with signals reproduced by density functional theory, resolving a decade-old puzzle.

## Graphical abstract

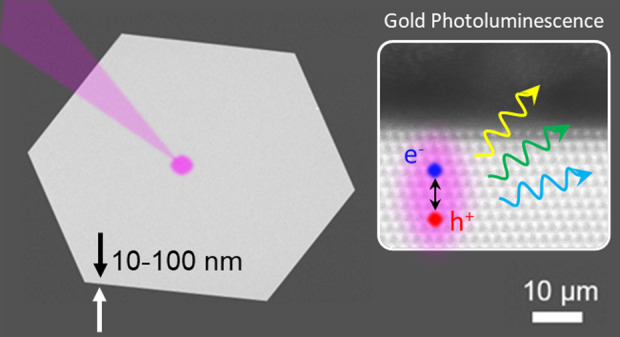

## Introduction

Luminescence from semiconductors following steady-state photoexcitation has been known since ancient times^1^. Today, semiconductor luminescence is widely employed as a non-invasive probe of diverse phenomena, including the dating of rocks, the assessment of solar cell efficiencies, and the monitoring of chemical reactions^2–5^. These applications are possible because the processes that cause luminescence from those materials are well understood. In contrast, luminescence from metals was first observed in 1969^6^, due to the signal being orders of magnitude weaker than in most semiconductors. Photon emission from metals has recently received increased attention in the context of plasmonic nanostructures, which promise to revolutionize industries including healthcare, sensing, and energy^7–9^ due to the ability of plasmon-generated hot carriers to dramatically elevate local electronic temperatures, enhance weak luminescence processes from molecules and increase solar cell absorption^10,11^. Steady-state luminescence has a unique potential to shed light on hot-carrier processes in plasmonic systems. Although it is more accessible from an experimental viewpoint, this process has received less attention than two-photon photoluminescence produced by pulsed lasers^12–15^. Nevertheless, steady-state luminescence from metals has been employed for fundamental nanoscale studies^16,17^, monitoring surface and electronic temperatures^18–21^, probing gold-molecule interactions^22,23^, and monitoring charge transfer processes^24,25^. Despite its usefulness, uncertainty remains around the origin of emitted light, particularly whether it is due to the recombination of electrons and holes (termed photoluminescence, PL) or other forms of inelastic light scattering, with many theoretical and experimental studies debating these possibilities over the last 50 years^14,17,26–33^ (see also a recent summary by Cai et al.^34^). This debate is further complicated by additional effects such as the Purcell enhancement of emission at specific wavelengths of light that resonate with plasmonic modes of the metal structure (which dominates the predicted spectrum in several works^17,35–40^), the position of the excitation wavelength relative to the interband transition threshold and spatial confinement. To the best of our knowledge, a full understanding of steady-state luminescence from metals following interband excitation without the participation of such resonant excitations is still lacking, thus hindering its applications as an effective probe.

Here, we study photon emission from 13 nm to 113 nm thick monocrystalline, atomically flat, gold flakes with the (111) surface exposed^41^. These samples allow us to probe the relationship between photon emission and nanoscale confinement without surface roughness or plasmonic enhancement, meaning that the conclusions we draw can be generally applied to any metal, not only those operating in the plasmonic regime. Our study reveals that, when illuminating in the interband regime, the long-wavelength photon emission is independent of the excitation wavelength, which is conclusive evidence that this signal is due to photoluminescence rather than other forms of inelastic scattering. In addition, we demonstrate that gold luminescence can be used as a probe of local temperature using only the Stokes signal (i.e., signal at longer wavelengths than the excitation wavelength) when exciting at 488 nm. To further understand the emission, we employ photon re-absorption to reveal that there is minimal charge diffusion after photoexcitation prior to photon emission. This enables us to present a model of luminescence that includes photon re-absorption and first-principles calculations based on density-functional theory (DFT), producing results in good agreement with PL experiments. Gold PL (when exciting in the interband regime) is shown to consist of two key components, both resulting from the recombination of excited d-band holes with unexcited electrons: pre-scattered luminescence close to the excitation energy, and longer wavelength post-scattered luminescence. Our model of bulk luminescence allows us to identify that, as the flake thickness is reduced below 40 nm, quantum-mechanical confinement of states near the Fermi level cause an increase in pre-scattered luminescence at longer wavelengths (when compared to thick flakes), which we justify via first-principles modeling. Finally, we explore luminescence signals when exciting in the intraband regime. By invoking scaling arguments, we propose that intraband luminescence is in fact not solely due to photoluminescence. Our results provide a comprehensive theory of gold photoluminescence in monocrystalline flakes that is readily applicable to other metals and nanoparticles. In addition, we realize a more accessible form of nanoscale thermometry and resolve a 50-year-old paradox on the origin of luminescence in gold.

## Results

**Fig. 1:**
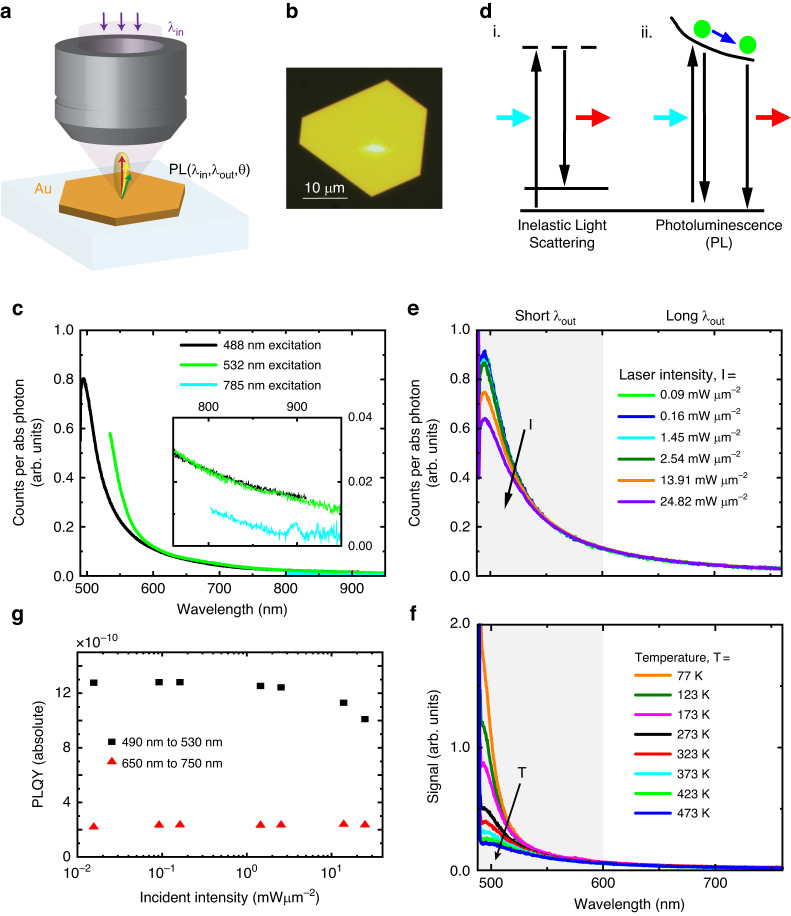
**a** Schematic of the measurement process including the incident laser spot and emitted light. **b** White light reflection image of a gold flake in a microscope (79 nm thickness), with a 488 nm wavelength laser incident on the central portion of the flake. **c** Photoluminescence of an 88 nm thick gold flake normalized per absorbed photon as a function of excitation wavelength. The inset expands the long wavelength region. Incident intensities are 1.449 mWμm^-2^/3.334 mWμm^-2^/0.063 mWμm^-2^ for 488 nm/532 nm/785 nm laser excitation wavelength. **d** Schematics of i. inelastic light scattering with no charge relaxation before luminescence and ii. photoluminescence processes. Photoluminescence involves the scattering of an excited electron (green dots), while non-photoluminescence inelastic light scattering is mediated by a virtual state (dashed line), without any relaxation process taking place prior to photon emission. **e**, **f** Spectral dependence of the photoluminescence of a 113 nm thick gold flake for **e** different laser excitation powers at room temperature, and **f** sample temperature for a constant excitation power of 0.072 mWμm^-2^. **g** External photoluminescence quantum yield (in absolute scale) as a function of excitation power for a 113 nm thick flake. In **e**–**g**, the excitation wavelength is 488 nm and the gray shaded area corresponds to the defined “short *λ*_out_” range

We synthetized bare monocrystalline gold flakes of 113 nm down to 13 nm thicknesses on quartz following Kiani et al.^41^ (with (111) surface exposed), with lateral dimensions greater than 5 μm in all samples. We studied 22 flakes and present representative results here. In all measurements, we used focused laser beams near the center of the atomically flat sample (i.e., far from edges to prevent plasmonic enhancement or the excitation of surface plasmons^17,42^). A schematic of the measurement and a white-light image of a gold flake also showing a focused laser spot on it are presented in Fig. 1a, b, respectively. To confirm that luminescence signals did not originate from defects or edges, we excited several points across the sample surface and found identical results. Additionally, we carried out spatially resolved measurements, which confirmed that signals originated solely from the region excited by the laser for all excitation wavelengths employed and excluded contributions from surface plasmons (see Supplemental Note 1). We also verified that the luminescence observed from our flakes was identical to monocrystalline gold fabricated by an entirely different synthesis method^43^ (Supplemental Note 2). Therefore, we are confident that our measurements present photon emission from gold itself without plasmonic enhancement effects being present, in contrast to most studies that specifically focus on the role of surface plasmons^44^. We also note that signals from flakes were unchanged over several months, demonstrating that our samples were stable.

We present photon emission from an 88 nm gold flake as a function of excitation wavelength in the interband (488 nm and 532 nm) and intraband (785 nm) regimes in Fig. 1c (signal normalized by the absorbed laser power). Broadband emission over the entire visible region is observed when exciting in the interband regime consistent with previous studies^6^. Interestingly, for the 785 nm laser excitation, the measured signal is weaker, but on the same order of magnitude, refuting previous reports that emission in this region is purely due to plasmonic effects^29,31^. We note the peak at 900 nm observed with 785 nm excitation is due to an experimental artifact—it is also observed on a silver mirror. When exciting in the interband regime (488 nm and 532 nm), we find that the emission spectra per absorbed photon overlap perfectly at long wavelengths, (i.e., *λ*_out_ ≳600 nm, Fig. 1c). This leads us to two important conclusions. Firstly, because the shape of the long wavelength emission spectrum is independent of the excitation wavelength, carriers must undergo relaxation prior to radiative recombination. Thus, a fast luminescence mechanism where there is no relaxation of excited charges before luminescence (as in Fig. 1d, i.), but where the emitted photon does not have the same energy as the incident photon (typically) due to coupling to phonons, can be ruled out in this regime. In support of this conclusion, we further note that signals do not overlap when plotted as a function of the energy shift relative to the incident laser energy (Supplemental Note 3). We can therefore conclude purely experimentally that photon emission at long wavelengths must be due to PL when exciting in the interband regime, as this allows for charge dynamics and relaxation prior to photon emission (Fig. 1d, ii.). Here, a photon triggers an electronic transition, and this electron can interact inelastically with other electrons and ions in the material before falling back to its ground state. Our result is in agreement with studies demonstrating that the lifetime of PL following interband excitation is on the order of 50 fs (while inelastic scattering is instantaneous)^45,46^. Secondly, as the absolute signals overlap in the long wavelength region, we can infer that carriers contributing to PL follow the same decay pathway, independent of excitation wavelength. In contrast, photon emission close to the excitation wavelength does not overlap when exciting with different lasers in the interband regime, consistent with previous observations of PL from gold nanoparticles^17^.

We varied the incident laser power on the sample to measure how the signal changed. For 488 nm excitation, we found the short wavelength emission (*λ*_out_ < 600 nm) PL reduced in magnitude (per absorbed photon) for higher incident laser powers (Fig. 1e, noting the absolute signal increases with laser power). Importantly, we do not observe a similar effect for 532 nm or 785 nm excitation (for comparable rates of absorbed photons). This change is fully reversible upon reducing the 488 nm laser power. We explain this behavior by noting that the same effect is observed upon sample heating (with fixed, low laser power), as is shown in Fig. 1f. By comparing the signal with laser irradiation and with external heating, we can measure the local sample temperature non-invasively for different laser powers, enabling a label-free thermometer for gold. We find that the lattice temperature can increase by ~200 K in thin samples (<20 nm) for the laser intensities used, while the lattice temperature only increases by 70 K for thick samples (>50 nm), as shown in Supplemental Note 4.

## Discussion

Considering the distinct behavior of the PL signal at short and long wavelengths for 488 nm excitation (with/without temperature dependence), we estimate the photoluminescence quantum yield (PLQY) separately for these two parts (Fig. 1g). Our external PLQY estimates of $\sim {10}^{-10}$ are in good agreement with Mooradian’s original estimates and well below those of plasmon-enhanced nanoparticle emission^6,47^. As reported before, apart from temperature effects, the PLQY remains constant when varying the excitation power^17^, so we are operating in the linear response regime. Importantly, this rules out nonlinear processes (for example, involving two excited carriers or the response of an already excited system, simplifying modeling^30^) and also implies that the signal is due to the recombination of an excited electron with an unexcited hole (or vice-versa). See Supplemental Note 5 and our previous work for further discussion^48^.

**Fig. 2:**
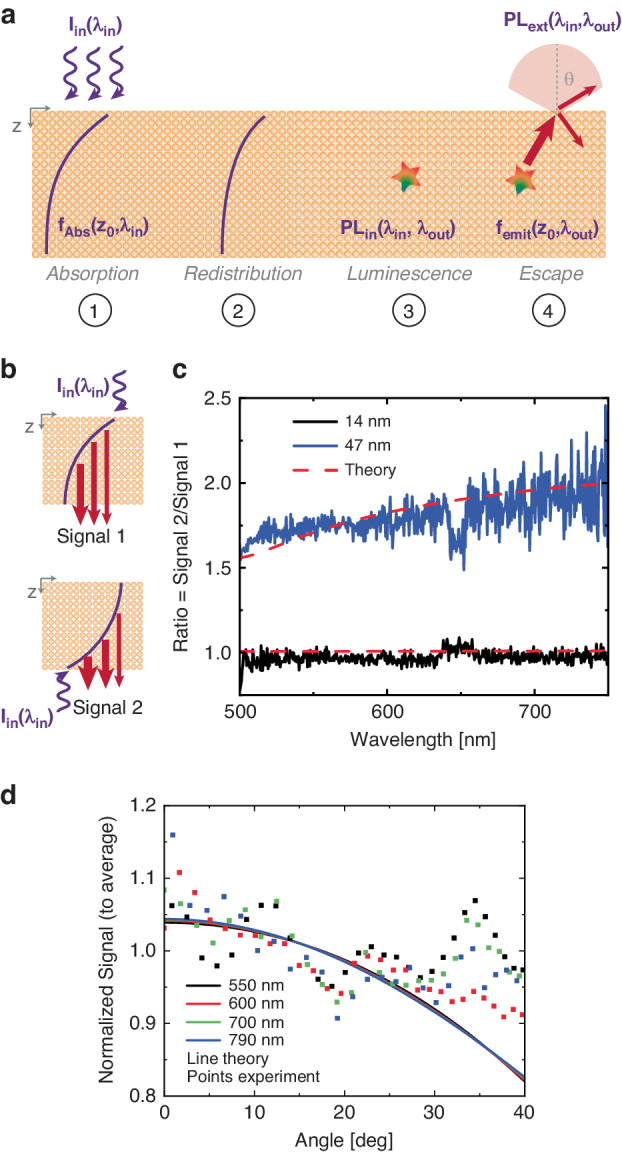
**a** Schematic of the processes that occur following photon absorption (equation 1), starting from point 1 and proceeding to point 4. b) Schematic of measurement approach—a gold flake is excited from above and below, and the photoluminescence signal is recorded from the same bottom side in both measurements (Signal 1 and Signal 2, respectively). **c** Wavelength dependence of the ratio between these two signals indicated in **b**, alongside calculations assuming that photoluminescence emission occurs from the locations where charges are generated. We present results for flakes of 47 nm and 14 nm thickness. For exciting and collecting the same (opposite) side, the excitation power is 0.057 mWμm^-2^ (0.052 mWμm^-2^). Signals are normalized to the number of incident photons. **d** Angle-resolved luminescence (normalized to the average value) at 550 nm, 600 nm, 700 nm, and 790 nm (±10 nm) emission wavelengths, alongside the predicted angular dependence, for 488 nm excitation on an 88 nm thick flake at 0.604 mWμm^-2^ excitation intensity

We modeled the luminescence from the gold using a formalism of dipoles emitting throughout the material^49^. As is schematically depicted in Fig. 2a, the measured PL is a combination of the dipole emission strength (based on the material’s band structure), charge transport prior to luminescence, and gold’s absorption coefficients. Regarding charge transport, we consider the two limiting cases of: (i) neglecting transport (i.e., excitation and emission take place at the same location); and (ii) a maximally delocalized model where equal emission occurs from every point inside the film. Within the far-field limit, and assuming that the luminescence spectrum is position independent, these models can be written as

1a $$P{L}_{{\rm{external}}}={{PL}}_{{\rm{internal}}}({\omega}_{\rm{in}},{\omega }_{{\rm{out}}})\left(\frac{{I}_{{{\rm{in}}}}}{{{\hslash }}{\omega }_{{\rm{in}}}}\right){\int_{0}^{d}}d{z}_{0}\,{f}_{{\rm{abs}}}\left({z}_{0},{\lambda }_{{\rm{in}}}\right)\,{f}_{{\rm{emit}}}\left({z}_{0},{\lambda }_{{\rm{out}}}\right)$$

for local PL and

1b $$P{L}_{{\rm{external}}}={\frac{{1}}{{d}}}\,P{L}_{{\rm{internal}}}\left({\omega }_{{\rm{in}}},{\omega }_{{\rm{out}}}\right)\left(\frac{{I}_{{\rm{in}}}}{{{\hslash }}{\omega }_{{\rm{in}}}}\right){\int_{0}^{d}}d{z}^{\prime}_{0}\, {f}_{{\rm{abs}}}\left({z}^{\prime}_{0} ,{\lambda }_{{\rm{in}}}\right){\int_{0}^{d}}d{z}_{0}\,{f}_{{\rm{emit}}}\left({z}_{0},{\lambda }_{{\rm{out}}}\right)$$

for maximally delocalized PL. Here, *PL*_external_ is the measured (external) luminescence per unit area and unit wavelength, *PL*_internal_ is the probability of photon emission at wavelength *λ*_out_ per photon absorbed at incident wavelength *λ*_in_ and unit of emission wavelength, $\frac{{I}_{{\rm{in}}}}{\hslash {\omega }_{{\rm{in}}}}$ is the rate of photons incident on the sample per unit area (expressed as the ratio of the laser intensity to the incident photon energy), ${f}_{{\rm{abs}}}\left({z}_{0},{\lambda }_{{\rm{in}}}\right)$ the fraction of incident photons absorbed per unit length across the film (noting that ${\int {f}_{{\rm{abs}}}\left({z}_{0},{\lambda }_{{\rm{in}}}\right){dz}}_{0}$ gives the total photon absorption in the material) and ${f}_{{\rm{emit}}}\left({z}_{0},{\lambda }_{{\rm{out}}}\right)$ describes the probability that a photon emitted at position *z*_0_ inside the metal escapes the material of thickness $d$ (encapsulating photon re-absorption processes) and is detected. We present a rigorous derivation of all terms in equation 1, parameterized by the refractive index of gold^50^, in Supplemental Note 6. Incidentally, the internal PL has been defined here to connect with the terminology used by the semiconductor luminescence community, though we note that care should be taken when attributing a physical meaning to this quantity, which is ultimately proportional to the dipole emission strength.

To model the luminescence according to equation 1, we need to (i) quantify the sample absorption and (ii) decide whether the local or delocalized model correctly describes the situation in gold. We performed reflection/transmission measurements to obtain the absorption spectra which are in agreement with theoretical calculations (see Supplemental Note 7 for absorption model and experimental results). As recently reported^51^, a way to spatially resolve the photon emission distribution is to measure the ratio of the PL spectra per absorbed photon when exciting the sample from the same and opposite sides as the signal collection (see schematic in Fig. 2b). In the case of localized PL, we should obtain a ratio significantly larger than 1 (that can be modeled using Eq. 1a). Conversely, for the maximally delocalized case a ratio of 1 is obtained for all metal film thicknesses. We present the measured and calculated ratios for 47 nm and 14 nm flakes in Fig. 2c. Strong support is obtained from the experiment to our local PL model, demonstrating minimal charge redistribution before PL, in agreement with theoretical predictions (which are in turn supported by recent experimental results^52,53^). This analysis also confirms that PL originates in the bulk of the film rather than from surfaces (which predicts significantly higher ratios, for example, the 47 nm thick flake presented ratios greater than 10, not shown on the plot). We find similar results for films of all thicknesses, though we note that for the thinner films (<25 nm) this ratio is close to 1 with or without charge motion because charges are more uniformly excited. We believe this is the first measurement revealing the spatial origin of gold luminescence.

To confirm our model of photon emission, we recorded the back-focal-plane (BFP) PL signal as a function of angle. A schematic of the emission configuration is presented in Fig. 2a (step 4). We plot the BFP PL signal for 550 nm, 600 nm, 700 nm, and 790 nm emission wavelengths from an 88 nm thick flake in Fig. 2d. We note this signal was extremely difficult to measure due to competing luminescence from the objective lens (which was much more strongly focused in BFP measurements and did not present a similar issue in real space measurements, see Methods). Despite this, on all plots, we observe agreement between the experiment and our prediction based on equation 1a.

**Fig. 3:**
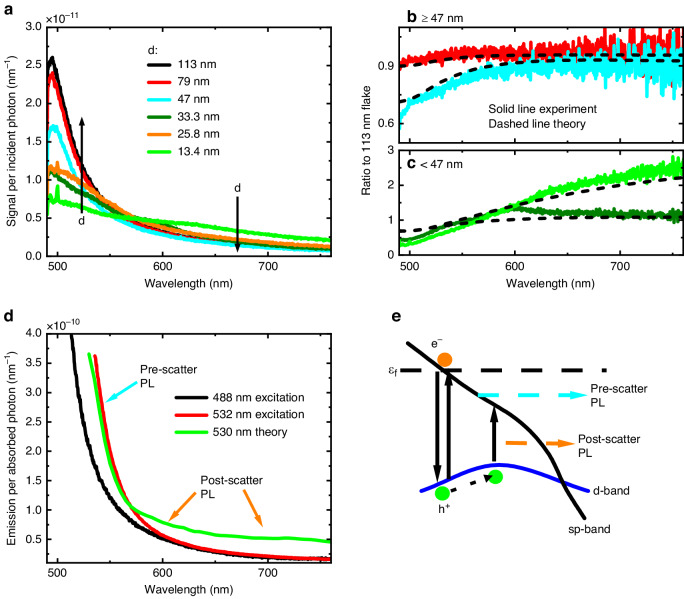
**a** Recorded photoluminescence per incident photon for different flake thicknesses *d* under 488 nm excitation wavelength. **b**, **c** Ratio of the measured signals presented in **a** to the signal obtained from the 113 nm flake and separated into **b** thick and **c** thin flakes, respectively. Dashed lines show calculated profiles based on the model presented in the main text (Eq. 2). Curve colors in **b**, **c** are coordinated with **a**. **d** Calculated internal luminescence (using an 88 nm thick flake data) for 488 nm and 532 nm light excitation, alongside simulated internal luminescence for 530 nm wavelength excitation. **e** Schematic of the processes leading to photoluminescence, where ε_f_ indicates the Fermi energy and h^+^/e^-^ represents the excited hole/electron following photoexcitation. The incident laser power on the sample is 0.162 mWμm^-2^/3.334 mWμm^-2^ for 488 nm/532 nm excitation wavelength in all panels

We now consider the role of film thickness in photon absorption and escape probability. Both quantities depend on thickness: for example, very thin films absorb much less light but the escape probability is much higher. We present the PL signal for gold microflakes with decreasing thicknesses (*d*) from 113 nm down to 13 nm in Fig. 3a. Two main changes occur: the short wavelength signal decreases, while the long wavelength signal increases. The sharp peaks at ~500 nm for thinner samples are quartz substrate Raman resonances. These changes can be due to changes in absorption and emission factors, or due to changes in the internal luminescence. To explore the contribution of *f*_abs_ and *f*_emit_ to signal changes with thickness, without having competing contributions from the spectral shape of *PL*_internal_, in Fig. 3b, c we present the ratio of the measured luminescence spectra relative to the signal measured for a 113 nm flake. In our model this is equivalent to the ratio:

2 $$\frac{{\int_{d}}\,{f}_{{\rm{abs}}}\,{f}_{{\rm{emit}}}{dz}}{{\int_{d=113{nm}}}\,{f}_{{\rm{abs}}}\,{f}_{{\rm{emit}}}{dz}}$$

This factor is overlaid as dashed lines in Fig. 3b, c for thick (≥47 nm) and thin (<47 nm) flakes, respectively. Good agreement is observed between experiment and theory down to 47 nm. However, for flakes thinner than approximately 40 nm, some deviations are observed: specifically, we record less signal at short wavelengths (<550 nm) and more signal at intermediate wavelengths (550 nm to 700 nm) than our model anticipates. However, theory and experiment still agree at sufficiently long wavelengths. Additionally, resonance features appear for thinner flakes, as can be observed in Fig. 3a. We first discuss flakes with thicknesses greater than 40 nm, which follows our theory well, and then explore thin flakes further.

We present *PL*_internal_ for 488 nm and 532 nm excitation in Fig. 3d, which we calculate by dividing the measured luminescence by the known factors in equations 1a. The internal PL is approximately an order of magnitude stronger than the measured PL (the wavelength resolved external PLQY is on the order of 10^-11^), and has a stronger signal at short wavelengths and weaker signal at long wavelengths when compared to the shape of the measured PL (see Supplemental Information Note 6). To better understand PL from gold, we developed a model parameterized by the output of DFT simulations that have already reproduced the optical constants of gold in the visible regime and finds quantitative agreement with pump-probe measurements on gold nanoparticle solutions^54–57^.

As Fig. 1d shows that long-wavelength luminescence is PL when exciting in the interband regime, we implemented a photoluminescence model^58–60^ that was first applied to metals by Apell et al.^61^ and more recently employed by several groups in experimental studies, notably Cai et al.^17^ (see Supplemental Note 8 for model details). Our model advances the literature by including both direct and phonon-assisted luminescence transitions, momentum conservation, and a steady-state excitation regime. We note that analytical expressions using an approach equivalent to ours have been applied in the case of intraband excitation^62^. Importantly, when computing PL, the complete non-thermal carrier population must be considered, including unscattered and scattered carriers. Our DFT-based luminescence simulations assumed charge carriers distributed across k-space (due to the difficulty in otherwise computing luminescence). However, this is demonstrably not the case immediately following interband photon absorption and before charge scattering, where charge carriers are at specific points in k-space. We derived a term to account for luminescence from pre-scattered carriers. Note that this is not necessary for modeling absorption in pump-probe spectroscopy because the pre-scattered carriers do not appreciably modify absorption at the lowest order, and hence the k-averaged treatment suffices there^56^. Due to lifetime broadening, unscattered carriers are expected to contribute to PL close to the excitation wavelength while scattered carriers produce PL at longer wavelengths (Supplemental Figure S12). We note that in intraband excitation, we expect unscattered PL (which is then phonon-assisted) to be negligible.

Our model is fully quantitative, allowing for a direct comparison of both spectra and intensity. We present simulated results for 530 nm excitation wavelength in Fig. 3d. We obtain good agreement between experiment and theory: luminescence decreases in intensity further from the excitation wavelength and becomes relatively flat at longer wavelengths. This plot is on an absolute, rather than a relative scale. The correct magnitude and spectral trend of the predicted signal confirm that luminescence close to the excitation wavelength is also photoluminescence (i.e., it is not mediated by a virtual state) and it is broadened due to the lifetime of the carrier in the real state. In Supplemental Figures S8 and S10 we show that, when exciting in the interband regime nearly all luminescence originates in d-band holes recombining with unexcited electrons (including emission at wavelengths longer than 700 nm—we note that, unlike absorption, interband PL can still occur in this region^63^). Therefore, due to the intensity and spectral separation of the emission from unscattered and scattered carriers, gold PL can be described as the sum of these two processes (Fig. 3e). To the best of our knowledge, this is the first time unscattered carrier luminescence has been shown to play a key role in steady-state luminescence from a material. We find a larger discrepancy between our experiment and theory at wavelengths longer than 600 nm, though well within the error for our experiments and calculations. This discrepancy, which could be due to additional loss processes in the metal not accounted for in simulations, or also to numerical artifacts, is discussed in Supplemental Note 8 section 5 alongside simulations with 488 nm excitation. We also discuss lattice temperature effects further, within the context of this model, in Supplemental Note 9^64^.

**Fig. 4:**
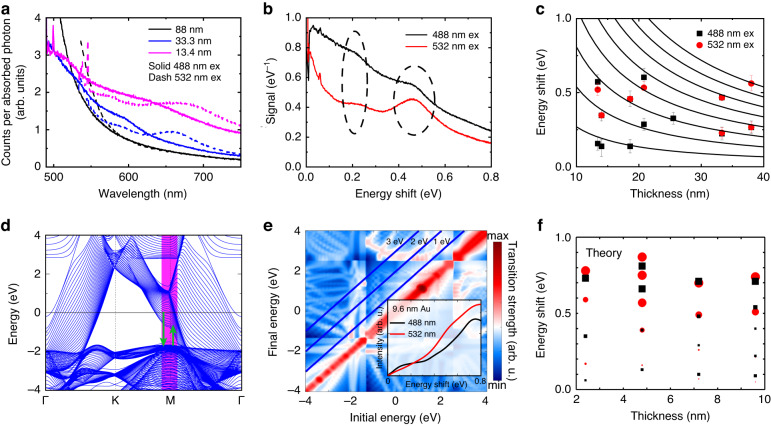
**a** Luminescence signal normalized per absorbed photon for flakes of 88 nm, 33.3 nm, and 13.4 nm thicknesses following excitation with 488 nm and 532 nm wavelength light (0.322 mWμm^-2^ and 0.549 mWμm^-2^ excitation intensities, respectively). **b** Luminescence signal for the 33.3 nm flake as a function of energy shift for 488 nm (1.449 mWμm^-2^) and 532 nm (3.334 mWμm^-2^) excitation wavelengths. **c** Energy shift of resonance peaks as a function of sample thickness for both 488 nm (black squares) and 532 nm (red circles), with the fitting described in the main text with different black lines corresponding to different n values, as marked on the figure. **d** Electronic band dispersions of 40 atomic layers of Au (111). The vicinity of the M point of the Brillouin zone is indicated by the pink area and the suggested pre-scattered luminescence process overlaid in green arrows, which indicate excitation and subsequent relaxation of a hole. **e** Dipole matrix elements of 40 atomic layers of Au (111) averaged over the pink area of the Brillouin zone shown in **d** as a function of initial and final state energies. Luminescence intensities estimated using the dipole matrix elements are shown in the inset as a function of energy shift for 488 nm and 532 nm excitation. **f** Variation of the predicted intensity (size of the black squares and red circles indicate the strength of the corresponding transitions emerging as features in the spectra of the inset in **e**) and energy shift of the resonance peaks obtained from first-principles calculations as a function of gold film thickness

We now discuss PL from ≤40 nm thick flakes. We present the PL signal per absorbed photon for several flake thicknesses for 488 nm and 532 nm excitation wavelengths in Fig. 4a. For thick flakes, the signal per absorbed photon with 488 nm and 532 nm excitation are identical at emission wavelengths (*λ*_out_) larger than approximately 600 nm (Fig. 1c and related discussion). However, the point where these signals start to overlap shifts to longer wavelengths as the flake thickness is reduced: for 33.3 nm it appears at 750 nm, while for 13.4 nm this overlap occurs at around 800 nm. Weak resonances are also observed for thin flakes (see Fig. 3a). Similar resonances were observed from such flakes in two-photon photoluminescence from thin flakes by Großmann et al.^13^. These resonances are inelastic (but before any electronic loss process), as can be seen in Fig. 4a: the resonance peak position shifts depending on the excitation wavelength. Resonance peaks are found to have a constant energy shift relative to the energy of the excitation wavelength, as shown in Fig. 4b for a 33.3 nm flake. We also observed comparable signals when exciting the film from the air side or through the quartz substrate (Supplemental Note 10), meaning these features can only be explained by changes to the electronic environment in the bulk material.

When we record the energy shift of the resonance peak (i.e., $\hslash ({\omega }_{{\rm{in}}}-{\omega }_{{\rm{out}}})$, with *ω*_out_ at the resonance peak) for different flake thicknesses (Fig. 4c), we find that, for a given thickness, energy shifts appear approximately at multiples of the lowest shift. For example, the energy shifts for a 33.3 nm flake are 0.23 eV and 0.46 eV, suggesting different orders of the same process. A plausible origin of the changes to luminescence lies in unscattered hole PL occurring at longer wavelengths, caused by the quantization of the band structure close to the Fermi level as sample thickness is reduced. This results in unscattered PL at multiples of the quantization spacing (see discussion in Supplemental Notes 11 and 12 and Figure S18 for a schematic of this process). Two additional points support this interpretation. Firstly, at sufficiently long wavelengths the signal still tends towards what we expect from our model presented in equation 1 (Fig. 3c), implying that the change is not due to a modification in the electron-electron or electron-phonon scattering rates (which are in any case local parameters), but rather to a change in the band structure experienced by excited holes. Secondly, we only observe these effects in ≤40 nm thick flakes. While the mean free path of holes in the d-band is only around 2 nm, the mean free path of a charge in a state near the Fermi level is around 40 nm^52^, indicating that these states are influenced by the finite thickness of the film. We find that different qualitative models can fit the data well (see Supplemental Note 11 for discussion). In Fig. 4c, we fit all data points by considering the breakdown of periodic boundary conditions in the vacuum direction (for electronic states close to the Fermi level). This gives energy shifts of $\frac{{nh}{v}_{f}}{2d}$, where *n* is an integer, *h* is Planck’s constant and *v*_*f*_ is the Fermi velocity (see Supplemental Note 11). We fit the experimental data well with ${v}_{f}=1.3\times {10}^{6}$ ms^-1^, close to the previous DFT values of $1.4\times {10}^{6}$ ms^-1^ ^52^.

We further support our claim that this effect is due to the breakdown of periodic boundary conditions with DFT calculations based on the thickness-dependent electronic band structure of Au (111) and the ensuing transition dipole matrix elements^65,66^. Electronic band dispersions of 40 layers of Au (111) are shown in Fig. 4d. As expected, the bands near the Fermi level are strongly quantized due to the finite thickness of the film and the lack of out-of-plane periodicity. The pink area around the M point of the Brillouin zone is highlighted as this is the relevant region that stands out as a candidate source of the experimentally observed spectral features originating in the piling up of many electronic bands around the Fermi level. We overlay green arrows (describing hole excitation and subsequent de-excitation) indicating the form of pre-scattered luminescence we believe to be responsible for the signals we observe. In Fig. 4e, the dipole matrix elements averaged over the area around the M points are shown as a function of the initial and final state energies. Transitions corresponding to 1, 2, and 3 eV energy jumps lie along the diagonal blue lines shown in the figure. Subsequently, using the formula given in Supplemental Note 12, we produce a rough estimate of the contribution of the dipole matrix elements to the luminescence intensity that occurs in the system when the material is illuminated with 488 nm and 532 nm excitation wavelength and illustrated it in the inset of Fig. 4e. It is worth noting that in our calculations, we collect all the transitions between the excitation and emission wavelength with equal energy for transitions around the M point. Additionally, we illustrate the resonance peaks in Fig. 4f (sizes of symbols show the relative strengths of the resonance amplitudes) as a function of thickness and optical energy shift. We find qualitative agreement between experimental and theoretical results: the energy shifts are of the same order of magnitude and we anticipate the same energy shifts for 488 nm and 532 nm excitation, especially for thicker flakes. Nevertheless, our calculations predict more resonance peaks at multiples of the lowest peak that are not revealed in the experiment, potentially due to the long wavelength signals being too weak for this to be measurable. Although the layer thicknesses that we consider in our calculations are smaller than in the experiment, it is clear that resonance peaks organized as a function of energy shift tend to overlap as the thickness increases, as also observed in the measurements.

Finally, we discuss the intraband luminescence (785 nm excitation, Fig. 1c). We apply an approach similar to that presented in Fig. 3c to explore how the signal changes with sample thickness (Supplemental Note 13). Importantly, when exciting at 785 nm, our model of how the signal changes with thickness agrees well for all samples, and we see no additional resonance features or significant deviations from theory (contrary to Fig. 3c for the thinner films). This is consistent with our interpretation of the resonances as pre-scattered photoluminescence: we predict pre-scattered luminescence to be much weaker than post-scattered luminescence for intraband transitions. We also calculate the internal intraband PL signal. Surprisingly, our DFT-parameterized model does not agree well with the experimentally recorded PL. Specifically, the experimental results are approximately a factor of five larger than those predicted by both the DFT-parameterized PL model and by simple scaling arguments, and the spectral profile of the luminescence is also significantly different. Therefore, our data demonstrates that, when exciting in the intraband regime, luminescence cannot be explained by photoluminescence alone. Instead, here we suggest that it receives contributions from other inelastic light scattering type processes. In Supplemental Figure S20d, we estimate the approximate magnitude of this effect based on our experiments, allowing future investigations to calculate its strength relative to PL. Our data suggests that photoluminescence alone is dominant when exciting in the interband regime, while there are additional processes in the intraband regime (which could well resemble those proposed by Hugall et al.^29^). While we note that some additional effects may change the relative contributions of PL and inelastic light scattering in nanoparticles (for example, geometry-assisted processes), our final insight contributes to resolve disagreements regarding the nature of gold luminescence—most studies claiming non-PL inelastic light scattering have used 785 nm excitation^29^, which corresponds to a photon energy below the interband threshold, while most studies claiming PL have employed interband excitation energies^17^.

In summary, we present a comprehensive study of photon emission from gold monocrystalline flakes following excitation with continuous wave lasers. We produce direct evidence from luminescence and present a first-principles parameterized model to show that this signal is consistent with photoluminescence when exciting in the interband regime. Importantly, we show that a significant component of photoluminescence arises from pre-scattered carriers. By comparing this model to data for different flake thicknesses, we find evidence of additional quantum mechanical effects that become important when the flakes are thinner than 40 nm, which we explain by noting that for thin flakes the electronic band structure changes near the Fermi level due to this thickness being comparable with the mean free path of Fermi-level electrons. Finally, by careful quantitative analysis based on our model, we propose that luminescence when exciting in the intraband regime cannot be explained alone by radiative emission associated with electron/hole recombination (i.e., PL), but rather has contributions arising from other inelastic light scattering-type processes (i.e., mediated by a virtual state^29^). We believe that our study offers a blueprint for studying luminescence in other metals besides gold, provides the first comprehensive model of continuous wave luminescence calculations in metals incorporating insight from density-functional theory, and reveals that quantum mechanical effects can emerge in the luminescence of metallic flakes below 40 nm thickness.

## Materials and methods

### Sample fabrication

Monocrystalline gold flakes were fabricated following Kiani et al. (using the bottom surface rather than the inter-substrate surface, see ref. ^41^). Here, samples were fabricated on quartz substrates (University Wafer) as glass photoluminescence was found to outcompete that from thin gold structures. Substrates were cleaned in ultrasonic baths of ethanol followed by de-ionized water (both for 15 min) prior to sample fabrication. Samples were washed in ethanol followed by de-ionized water, and then dried with a nitrogen gun, before any measurements.

### Luminescence measurements

488 nm laser excitation measurements presented in Figs. 1d–f, 4a and Supplemental Figs. S2–4, and all 532 nm laser excitation measurements were carried out on Renishaw inVia Raman Microscope RE04, for 488 nm using a Coherent sapphire 488 SF NX and 532 nm Nd-YAG (RL532100) excitation and a Leica HC PL FLUOTAR 100x long working distance objective (NA = 0.75). All other measurements were carried out on a NanoMicroSpec-Transmission™ (NT&C) microscope, further adapted to enable Raman spectroscopy (which gave excellent agreement with the commercial Renishaw system). Specifically, 488 nm (Matchbox) and 785 nm (Thorlabs L785H1 diode mounted into a Thorlabs LDM56/M, current and temperature controlled by LDC205C and TED200C) continuous-wave lasers were coupled into a Nikon Eclipse Ti2 inverted microscope. Here the objective was a 60x Nikon S Plan Fluor (NA = 0.7). The laser beam was passed through laser line bandpass filters (Thorlabs FL488-1 and Semrock LL01-488 for 488 nm excitation/Semrock LL01-785 for 785 nm excitation) prior to impacting the sample. For both excitation wavelengths two Thorlabs notch filters (NF488-15/NF785-33 for 488 nm and 785 nm excitation) were used to remove any laser from the collected signal. The signal was coupled to a Princeton Instruments Spectra Pro HRS-500 spectrometer and recorded on a PIXIS 256 camera. All signals were radiometrically calibrated using a calibration lamp (Ocean Optics HL-3P-INT-CAL) coupled to an integrating sphere (Thorlabs 2P3/M), see ‘photoluminescence quantum yield’ section for further details. Spot sizes were assumed diffraction limited on the Renishaw inVia Raman Microscope. For the NT&C system, the laser spot size was recorded using a camera and fitted by a two-dimensional Gaussian. Spot size here is as the intensity that falls to $\frac{1}{{e}^{2}}$ of the maximum intensity. When calculating the relative emission intensity of signals with different laser excitation wavelengths (as in Figs. 1c and 4a) we first performed measurements using one laser and then carried out a calibration measurement. Without removing the lamp or the calibration sphere, we then changed the collection path filters for measurements with a second laser, and we then recorded the calibration on this adjusted setup. This means that even if our PLQY estimates contain inaccuracies, the relative intensity measurements of different laser excitation wavelengths is robust.

### Laser power determination

was recorded using a Thorlabs S170C or S130C power meter (using the microscope power meter for any measurements at the sample position).

### Back-focal-plane

Two lenses were placed between the image plane of the microscope and the spectrometer to enable back-focal-plane measurements, following methods described by Kurvits et al.^67^. Initially, a signal of a larger order of magnitude than the gold signal was observed when no sample was present in the system, which we attribute to the objective lens glowing. To remove this objective signal from BFP measurements we closed an iris in a real space plane between the sample and detector, to exclude signals except those originating from the region surrounding the sample. However, we still observed a small background signal from the objective. The objective signal was measured when no sample was present, and this objective signal multiplied by 2.7 was subtracted from the sample signal. The value of 2.7 was calculated from ${Objective\; signal}\times \left(1+R\left({\lambda }_{{\rm{out}}}\right)+R\left({\lambda }_{{\rm{in}}}\right)+R\left({\lambda }_{{\rm{out}}}\right)\times R\left({\lambda }_{{\rm{in}}}\right)\right)$, where *R* is the power reflection coefficient of the sample. This originates from the objective signal being reflected from the sample, plus the reflected laser causing the objective to fluoresce a second time when compared to the case of no sample present. We note this correction also removed the angular profile of the objective glow from our measurement. Lastly, we note that in real-space measurements the objective glow was orders of magnitude weaker than our sample signal, due to the sample signal originating from one small region in real space.

### Photoluminescence quantum yield (PLQY)

was estimated using a similar approach to that outlined by Frohna et al.^68^. Specifically, a calibrated light source (Ocean Optics HL-3P-INT-CAL) was coupled to an integrating sphere with a known spectral response (Thorlabs 2P3/M). The output port of this sphere was aligned with the objective lens of the Raman microscope and the spectrum was recorded (allowing for relative radiometric calibration). The notch filter that normally removes the laser signal from the recording path was then removed, and the signal was again recorded. The ratio of these signals gives the spectral response with and without the notch filter present. Secondly, the sphere was removed and the objective lens focused on a mirror (Thorlabs PF10-03-P10-10) with a known spectral response. The laser normally used to excite the sample was then shone on the mirror (at low power) and the signal was recorded on the spectrometer. Finally, the mirror is removed and the incident power at the same position is recorded. By accounting for the reflection strength of the mirror, the ratio of the number of photons incident on the mirror and the number recorded by the spectrometer can be calculated (i.e., an absolute calibration at one wavelength). Using the recorded lamp spectrum, it is then possible to work out an absolute calibration at any wavelength both with the notch filter present and removed. This calibration allows for PLQY measurement. We note that the relative radiometric response of the sphere was measured by sending a collimated white light beam into a second integrating sphere and recording the signal at the output port on an Ocean Insight Spectrometer FLAME-S-XR1. The sphere of interest was then coupled to the second integrating sphere and the collimated beam was sent into the sphere of interest. The ratio of these two signals gives the spectral response of the integrating sphere used in measurements.

### Absorption measurements

Absorption measurements were recorded on the NT&C system (a Nikon Eclipse Ti2 coupled to a Princeton Instruments Spectra Pro HRS-500 spectrometer and a PIXIS 256 camera). The light source was an Energetica LDLS^TM^ laser-driven white light source output through a fiber with 100 μm core diameter. For transmission, incident light was collimated and then focused on the sample through a condenser lens with variable a-stop (almost fully closed), while for reflection incident light was focused in the center of the objective back focal plane. In both cases this allowed light to be incident perpendicular to the sample. By appropriate choice of lenses for transmission, and by use of an iris for reflection, the illuminated area was reduced to approximately 4 μm^2^ (i.e., smaller than the size of any gold flake). All recorded signals were passed through a 450 nm long pass filter (Thorlabs FELH0450) to prevent second-order effects. For transmission, a bare quartz substrate was used as a reference (included in the modeling), while for reflection a mirror of known spectral response (Thorlabs PF10-03-P10-10) was employed as the reference (the Thorlabs reported spectral response was used). When calculating the signal per absorbed photon, the modeled absorption values were used (though we note extremely similar values are obtained using experimental values).

### Atomic force microscopy (AFM)

was used to measure sample thicknesses. A Bruker FastScan AFM was employed for all measurements in ScanAsyst mode. ScanAsyst-Fluid+ tips were used in all measurements. 0^th^ order flattening and 1^st^ order plane fits were applied to all results.

### Supplementary information

Supplemental Material

## Data Availability

The data underlying this manuscript are available at https://zenodo.org/doi/10.5281/zenodo.10654375.
