# Supplementary material for: Quantum-mechanical effects in photoluminescence from thin crystalline gold films
Source: Light Sci Appl. 2024 Apr 19;13:91. doi: 10.1038/s41377-024-01408-2 (PMC11026419; doi:10.1038/s41377-024-01408-2)
Supplement: Supplementary file 1 — Supplemental Material [file 41377_2024_1408_MOESM1_ESM.pdf]

Supplemental Information for Quantum-mechanical effects in photoluminescence from thin crystalline gold films

Alan R. Bowman[1], Alvaro Rodríguez Echarri[2,3], Fatemeh Kiani[1], Fadil Iyikanat[2], Ted V. Tsoulos[1], Joel D. Cox[4,5], Ravishankar Sundararaman[6,7], F. Javier García de Abajo[2,8], and Giulia Tagliabue[1]*

1. Laboratory of Nanoscience for Energy Technologies (LNET), STI, École Polytechnique Fédérale de Lausanne (EPFL), Lausanne 1015, Switzerland
2. ICFO–Institut de Ciencies Fotoniques, The Barcelona Institute of Science and Technology, 08860 Castelldefels (Barcelona), Spain
3. MBI–Max-Born-Institut, 12489 Berlin, Germany
4. POLIMA–Center for Polariton-driven Light–Matter Interactions, University of Southern Denmark, Campusvej 55, DK-5230 Odense M, Denmark
5. Danish Institute for Advanced Study, University of Southern Denmark, Campusvej 55, DK-5230 Odense M, Denmark
6. Department of Materials Science & Engineering, Rensselaer Polytechnic Institute, 110 8th Street, Troy, New York 12180, USA
7. Department of Physics, Applied Physics, and Astronomy, Rensselaer Polytechnic Institute, 110 8th Street, Troy, New York 12180, USA
8. ICREA–Institució Catalana de Recerca i Estudis Avançats, Passeig Lluís Companys 23, 08010 Barcelona, Spain

*corresponding author: giulia.tagliabue@epfl.ch

Supplemental Note 1 – Spatial resolution of flake emission

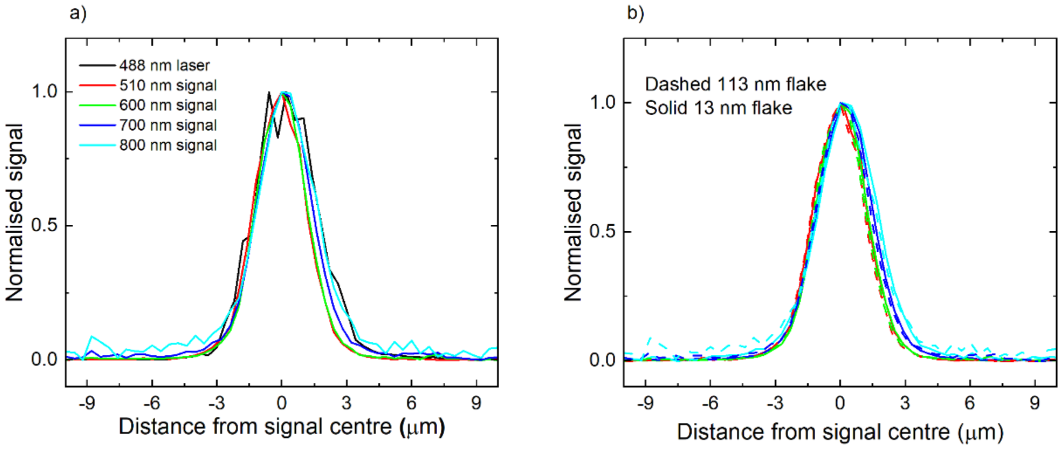

Figure S1. a) In-plane spatial spread of the photoluminescence signal compared to the spread of the laser beam for a flake of 113 nm thickness. b) Comparison of signal spread for 113 nm and 13 nm thickness flakes. In all measurements, the 488 nm laser intensity used here is 0.079 mWμm^{-2}, and the legend in a) applies to both plots.

In Figure S1a, we superimpose the spatially resolved emission profile from a gold flake and the laser spot shape on the surface of the sample. The two are in strong agreement, showing that the signal only originates from the position where the laser is acting. Different flakes are of different lateral sizes, with the smallest lateral size being for our 13 nm flake. In Figure S1b we compare the signal spread of the 113 nm and 13 nm flakes and find that, within experimental error, they agree. We note that, when processing our measurements (which were spatially resolved when we used our home-built system, see methods), we only processed signals from the region surrounding the laser spot rather than the full spectrometer array (removing any signal from the objective glowing or similar). We saw no effects of surface plasmons at flake edges (for further discussion of surface plasmon effects more generally see reference [1]).

Supplemental Note 2 – Comparing the emission from samples fabricated from different synthesis methods

In Figure S2, we present the photoluminescence from a 113 nm gold flake synthesised by Kiani et al.'s method [2] and a 200 nm commercial flake on a mica substrate (note the gold is thick enough so the substrate does not affect the optical response) fabricated by Phasis [3], illuminated with the same laser intensity. The crystallographic orientation of the exposed surface is (111) in both flakes. The signals are identical, confirming that our observations do not depend on the synthesis method.

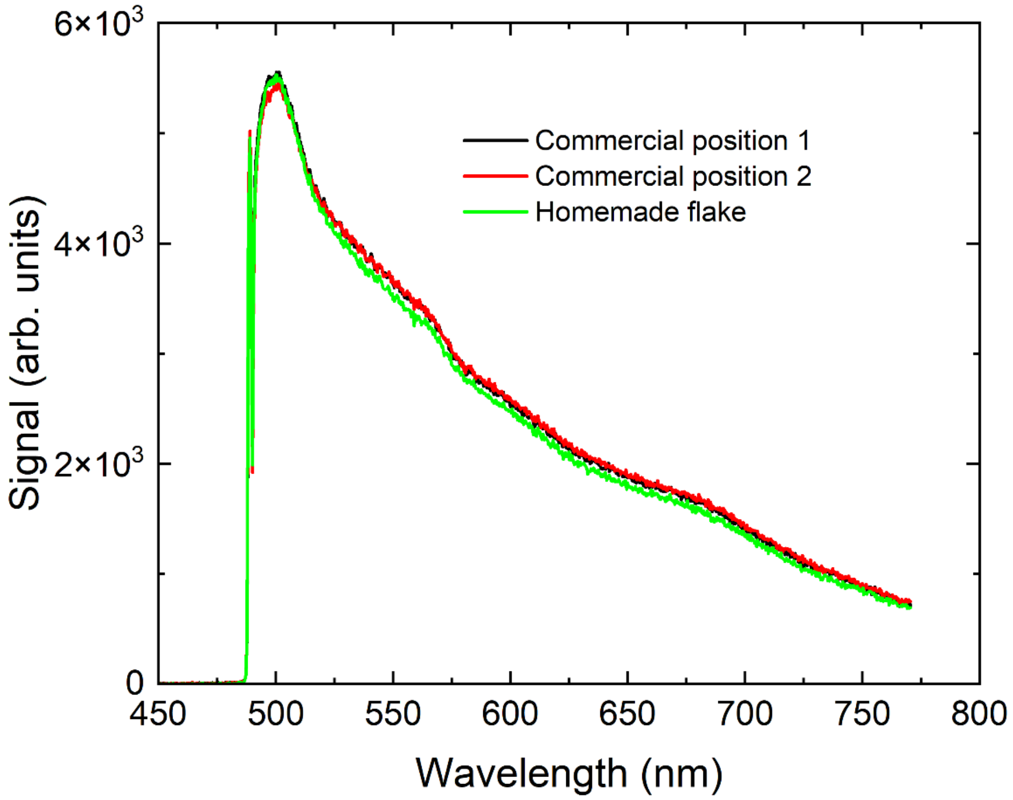

Figure S2. Comparison of photoluminescence signals from a 113 nm flake fabricated by Kiani et al.'s method (green curve) and a 200 nm commercially available monocrystalline flake measured in two different positions (black and red curves) when excited by a 488 nm laser (0.042 mWµm^{-2} intensity). We note that these signals are not radiometrically calibrated.

Supplemental Note 3 – Long-wavelength signals do not overlap in energy shift

In Figure S3, we present the results from Figure 1a reformatted to show the signal as a function of energy shift relative to the incident laser intensity. When presented as energy shifts, the signals no longer overlap.

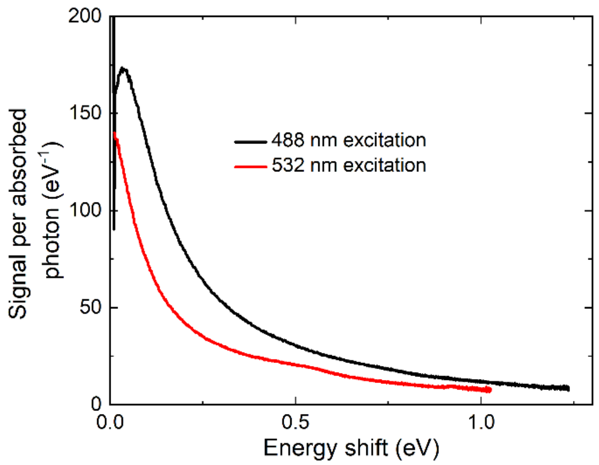

Figure S3. Signal per absorbed photon for a 113 nm flake as a function of energy shift for 488 nm and 532 nm excitation wavelengths. The light intensity is 1.449 mWμm^{-2}/3.334 mWμm^{-2} for 488 nm/532 nm excitation.

Supplemental Note 4 – Linear scaling of the local sample temperature with laser excitation intensity

In Figure S4, we present the temperature extracted from the flake as a function of the absorbed laser power for 14 nm and 113 nm gold thicknesses. We find that thinner flakes reach higher temperatures for the same absorbed intensity, as expected from the increased thermal confinement. We note that there is a larger uncertainty in the temperature measurement for the thinner flake as the cryostat sample stage also contributes a weak temperature-dependent Raman signal that competes more strongly with the signal emanating from thinner flakes. We measured this stage contribution separately and subtracted it from the gold signal, but some uncertainty remains.

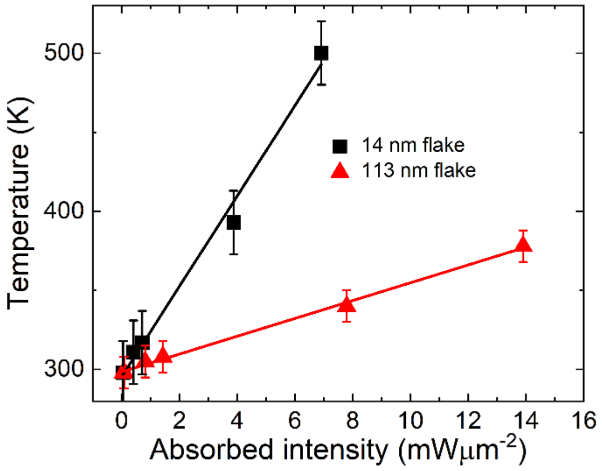

Figure S4. Measured sample temperature as a function of absorbed laser power, as calculated from the measured laser power and the calculated absorption based on analysis in Supplemental Note 7. We show results for 14 nm and 113 nm flakes excited by 488 nm light.

Supplemental Note 5 – Scaling of photoluminescence quantum yield with excitation intensity

We can define the external PLQY as

$$PLQY_{\mathrm{ext}} = \frac{Photons\ out}{Photons\ absorbed} = \frac{Radiative\ recombination\ rate}{Total\ generation\ rate, G}.$$

As we are operating in a steady state regime (at the excitation densities quoted more than 1 photon is being absorbed in the material every fs) and considering that the emitted photons we measure satisfy $\hbar\omega_{\mathrm{out}} > \frac{\hbar\omega_{\mathrm{in}}}{2}$, the generation rate is proportional to the total recombination rate, that is, $G \propto R$. Furthermore, in metals, at low excitation density (as is the case in continuous wave measurements), $R \propto n_{\mathrm{ex}} = p_{\mathrm{ex}}$, that is, the recombination rate scales linearly with the total number of excited electrons (n_{ex}), which is in turn equal to the number of excited holes (p_{ex}, to preserve charge neutrality, and assuming a minimal effect of charge traps). This is because, at low excitation density, both electron-electron and electron-phonon scattering are linear in the number of excited charge carriers and generation of additional electron-hole excited pairs thus scales linearly with the initial number of excited charge carriers.

In general, the radiative recombination rate is $\propto Ap_{\mathrm{ex}}n_{\mathrm{ex}} + Bn_{\mathrm{ex}} + Cp_{\mathrm{ex}}$, where the first term corresponds to recombination of excited electrons with excited holes, while the second and third terms correspond to the radiative recombination of excited electrons/holes with unexcited carriers, respectively. This expression involves constant coefficients A, B and C. Combining the above expressions, we find

$$PLQY_{\mathrm{ext}} \propto AG + D,$$

where G is proportional to the incident laser power and $D = B + C$. As we observe in Figure 1d, $PLQY_{\mathrm{ext}}$ is constant as the laser power increases. Therefore, the recombination of excited charge carriers with unexcited charge carriers is dominant.

Supplemental Note 6 – Electromagnetic theory of photoluminescence produced upon radiative recombination

Electron-hole recombination events involve relatively small distances compared to the light wavelength, and therefore, we assimilate the emission to that of localized dipolar emitters. The actual strength and number of those emitters depend on the electron dynamics following laser irradiation, as discussed below. However, the electromagnetic aspects of the emission can be safely described by formulating a semi-analytical theory under the assumption of the localized-dipole-emitters model combined with the local response model (i.e., the involved materials are represented through their frequency-dependent local permittivities). Next, we present a derivation of the emission intensity produced by a dipolar emitter placed inside the metal film (see Figure S5) based on the solution of Maxwell's equations, which is expressed in terms of the electromagnetic Green tensor. As the measured signal originates from the region surrounding the laser spot (Supplemental Note 1), we do not consider surface plasmons out-coupling at flake edges in our model. In this Supplemental Note, we work in Gaussian units. We consider a thin metallic film with permittivity $\epsilon_{\rm m}$ and thickness d that spans the region $z = 0$ to $z = d$ and interfaces a homogeneous superstrate (substrate) with permittivity ϵ_1 (ϵ_3), as schematically illustrated in Figure S5.

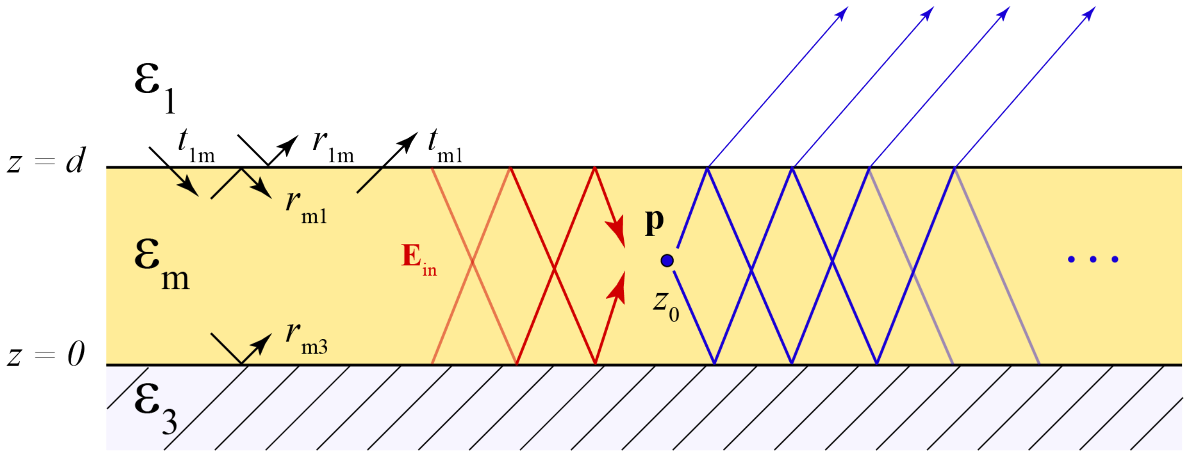

Figure S5. Illustration of a dipole emitting inside a metallic film. A dipole $\mathbf{p}$*, which is excited by an impinging field* $\mathbf{E}^{\rm exc}$ *(red lines), is placed at* $z = z_0$ *inside a film of thickness* d *and permittivity* $\epsilon_{\rm m}$ *adjacent to homogeneous media with permittivity* ϵ_1 *above and* ϵ_3 *below. The dipole generates waves (blue lines) that undergo multiple reflections before they escape to the far field, where the emission is eventually detected.*

1. Electromagnetic theory

To compute the electric field outside the film, we follow a well-establish procedure [4] and start with the field produced at a position $\mathbf{r} = (\mathbf{R}, z)$, with $\mathbf{R} \equiv (x, y)$, by a dipole $\mathbf{p}$ located at $\mathbf{r}_0$ inside a homogeneous bulk metal [5],

$$\mathbf{E}^{\rm dip}(\mathbf{r},\omega) = \frac{1}{\epsilon_{\rm m}}(k_{\rm m}^2 + \nabla\times\nabla)\,\mathbf{p}\frac{e^{ik_{\rm m}|\mathbf{r}-\mathbf{r}_0|}}{|\mathbf{r}-\mathbf{r}_0|},$$

where the wavenumber $k_{\rm m} = k\sqrt{\epsilon_{\rm m}}$ in the metal is expressed in terms of the vacuum wavenumber $k = \omega/c$ and ω is the emission frequency. Using the identity

$$\frac{e^{ik_{\rm m}|\mathbf{r}-\mathbf{r}_0|}}{|\mathbf{r}-\mathbf{r}_0|} = \int \frac{d^2\mathbf{Q}}{(2\pi)^2}\frac{2\pi i}{k_{\rm mz}}\, e^{i\mathbf{Q}\cdot(\mathbf{R}-\mathbf{R}_0)}\, e^{ik_{\rm mz}|z-z_0|},$$

in which we have introduced the in-plane wave vector $\mathbf{Q} = (Q_x, Q_y)$ and the normal component $k_{\rm mz} = \sqrt{k_{\rm m}^2 - Q^2}$ (with the square root taken such that $\mathrm{Im}\{k_{\rm mz}\} > 0$ and Q being the magnitude of $\mathbf{Q}$), along with the unit vectors $\hat{k}_{\rm m}^{\pm} = \frac{\mathbf{Q}\pm k_{\rm mz}\hat{z}}{k_{\rm m}}$, $\mathbf{e}_{\rm s} = \frac{-Q_y\hat{x}+Q_x\hat{y}}{Q}$, and $\mathbf{e}_{\rm p,m}^{\pm} = \frac{\pm k_{\rm mz}\mathbf{Q}-Q^2\hat{z}}{Qk_{\rm m}}$ forming a complete and orthonormal set, the dipole field becomes

$$\mathbf{E}^{\rm dip}(\mathbf{r},\omega) = \frac{ik^2}{2\pi}\int\frac{d^2\mathbf{Q}}{k_{\rm mz}}\left[(\mathbf{e}_{\rm s}\cdot\mathbf{p})\,\mathbf{e}_{\rm s} + \left(\mathbf{e}_{\rm p,m}^{\pm}\cdot\mathbf{p}\right)\mathbf{e}_{\rm p,m}^{\pm}\right]e^{i\mathbf{Q}\cdot(\mathbf{R}-\mathbf{R}_0)}e^{ik_{\rm mz}|z-z_0|}.$$

In the metal film, the generated electromagnetic s- and p-waves travelling up and down undergo reflections at the interfaces. Taking interface scattering into account, the electric field above the film (i.e., in medium 1 outside the metal film; see Figure S5), where we measure the luminescence signal, is given by

$$\mathbf{E}_{\rm m}(\mathbf{r},\omega) = \frac{ik^2}{2\pi}\int\frac{d^2\mathbf{Q}}{k_{\rm mz}}\, e^{i\mathbf{Q}\cdot(\mathbf{R}-\mathbf{R}_0)}e^{ik_z|z-z_0|}$$

$$\times\begin{Bmatrix} \mathbf{e}_{\rm p}^{+}\left[\left(\mathbf{e}_{\rm p,m}^{+}\cdot\mathbf{p}\right)\dfrac{t_{\rm p,m1}e^{ik_{\rm mz}(d-z_0)}}{1-r_{\rm p,m1}r_{\rm p,m3}e^{2ik_{\rm mz}d}} + \left(\mathbf{e}_{\rm p,m}^{-}\cdot\mathbf{p}\right)\dfrac{r_{\rm p,m3}t_{\rm p,m1}e^{ik_{\rm mz}(d+z_0)}}{1-r_{\rm p,m1}r_{\rm p,m3}e^{2ik_{\rm mz}d}}\right] \\ \mathbf{e}_{\rm s}(\mathbf{e}_{\rm s}\cdot\mathbf{p})\dfrac{t_{\rm s,m1}}{1-r_{\rm s,m1}r_{\rm s,m3}e^{2ik_{\rm mz}d}}\left[e^{ik_{\rm mz}(d-z_0)} + r_{\rm s,m3}e^{ik_{\rm mz}(d+z_0)}\right]\end{Bmatrix}, \qquad \text{(S1)}$$

where we introduce Fresnel transmission and reflection coefficients from the metal to the air/glass as [4]

$$r_{\rm s,m1} = \frac{k_{\rm mz}-k_{1z}}{k_{\rm mz}+k_{1z}}, \qquad t_{\rm s,m1} = \frac{2k_{\rm mz}}{k_{\rm mz}+k_{1z}},$$

$$r_{\rm p,m1} = \frac{\epsilon_1 k_{\rm mz}-\epsilon_{\rm m}k_{1z}}{\epsilon_1 k_{\rm mz}+\epsilon_{\rm m}k_{1z}}, \qquad t_{\rm p,m1} = \frac{2\sqrt{\epsilon_{\rm m}\epsilon_1}k_{\rm mz}}{\epsilon_1 k_{\rm mz}+\epsilon_{\rm m}k_{1z}}.$$

We can find an expression for the electric field in the far field (FF) in air that takes the form $\mathbf{E}^{\rm FF} = \boldsymbol{f}(\hat{\boldsymbol{r}})\frac{e^{ikr}}{r}$ by taking the limit $kr\to\infty$ in the expression of the electric field $\mathbf{E}_{\rm m}(\mathbf{r},\omega)$, where we apply the identity

$$\lim_{kr\to\infty}\int\frac{d^2\mathbf{Q}}{(2\pi)^2}\frac{2\pi i}{k_z}e^{i\mathbf{k}\cdot\mathbf{r}}g(\mathbf{Q}) = \left[g(\mathbf{Q})\frac{e^{ikr}}{r}\right]_{\mathbf{Q}=k\frac{\mathbf{R}}{r}}$$

for a given kernel $g(\mathbf{Q})$. The left-hand side of this equation has the same structure as Eq. (S1), from which we obtain an explicit expression of the kernel. We thus find

$$|\boldsymbol{f}(\hat{\boldsymbol{r}})|^2$$
$$= \left|\mathrm{e}^{-\mathrm{i}k_z d}\right|^2 \left|\frac{k^2 k_z}{k_{\mathrm{mz}}}\right|^2 \left\{ \begin{array}{c} \left| \left(\mathbf{e}_{\mathrm{p,m}}^{+} \cdot \mathbf{p}\right) \frac{t_{\mathrm{p,m1}} \mathrm{e}^{\mathrm{i}k_{\mathrm{mz}}(d-z_0)}}{1 - r_{\mathrm{p,m1}} r_{\mathrm{p,m3}} \mathrm{e}^{2\mathrm{i}k_{mz}d}} + \left(\mathbf{e}_{\mathrm{p,m}}^{-} \cdot \mathbf{p}\right) \frac{r_{\mathrm{p,m3}} t_{\mathrm{p,m1}} \mathrm{e}^{\mathrm{i}k_{\mathrm{mz}}(d+z_0)}}{1 - r_{\mathrm{p,m1}} r_{\mathrm{p,m3}} \mathrm{e}^{2\mathrm{i}k_{\mathrm{mz}}d}} \right|^2 \\ \left| \left(\mathbf{e}_{\mathrm{s}} \cdot \mathbf{p}\right) \frac{t_{\mathrm{s,m1}}}{1 - r_{\mathrm{s,m1}} r_{\mathrm{s,m3}} \mathrm{e}^{2\mathrm{i}k_{\mathrm{mz}}d}} \left[\mathrm{e}^{\mathrm{i}k_{\mathrm{mz}}(d-z_0)} + r_{\mathrm{s,m3}} \mathrm{e}^{\mathrm{i}k_{\mathrm{mz}}(d+z_0)}\right] \right|^2 \end{array} \right\}.$$

We now assume incoherent dipoles of equal weight oriented along x, y, and z directions with magnitudes $p_x = p_y = p_z \equiv p$. The corresponding projections of the unit vectors on Cartesian axes read

$$\mathbf{e}_{\mathrm{s}} \cdot \hat{\boldsymbol{x}} = -\frac{Q_y}{Q}, \qquad \mathbf{e}_{\mathrm{s}} \cdot \hat{\boldsymbol{y}} = \frac{Q_x}{Q}, \qquad \mathbf{e}_{\mathrm{s}} \cdot \hat{\boldsymbol{z}} = 0,$$

and

$$\mathbf{e}_{\mathrm{p,m}}^{\pm} \cdot \hat{\boldsymbol{x}} = \pm \frac{Q_x k_{\mathrm{mz}}}{Q k_{\mathrm{m}}}, \qquad \mathbf{e}_{\mathrm{p,m}}^{\pm} \cdot \hat{\boldsymbol{y}} = \pm \frac{Q_y k_{\mathrm{mz}}}{Q k_{\mathrm{m}}}, \qquad \mathbf{e}_{\mathrm{p,m}}^{\pm} \cdot \hat{\boldsymbol{z}} = -\frac{Q}{k_{\mathrm{m}}}.$$

This allows us to write the total far-field intensity and averaging over the three Cartesian dipole orientations as

$$|\boldsymbol{f}(\hat{r}, z_0)|^2 = \left|\mathrm{e}^{-\mathrm{i}k_z d}\right|^2 k^4 p^2 \left\{ \left|\frac{k_{\mathrm{mz}}}{k_{\mathrm{m}}}\right|^2 D_{\mathrm{p}}^{-} + D_{\mathrm{s}}^{+} + \left|\frac{Q}{k_{\mathrm{m}}}\right|^2 D_{\mathrm{p}}^{+} \right\},$$

where

$$D_{\sigma}^{\pm}(\theta, z_0) = \left| \frac{t_{\sigma,1\mathrm{m}}}{1 - r_{\sigma,\mathrm{m1}} r_{\sigma,\mathrm{m3}} \mathrm{e}^{2\mathrm{i}k_{\mathrm{mz}}d}} \left[\mathrm{e}^{\mathrm{i}k_{\mathrm{mz}}(d-z_0)} \pm r_{\sigma,\mathrm{m3}} \mathrm{e}^{\mathrm{i}k_{\mathrm{mz}}(d+z_0)}\right] \right|^2$$

depends explicitly on the dipole depth position z_0 and the emission angle θ, and we have used the identity $\left|\frac{k_{1z} t_{\sigma,\mathrm{m1}}}{k_{\mathrm{mz}}}\right|^2 = \left|t_{\sigma,1\mathrm{m}}\right|^2$, with the subscript 1m indicating transmission from air to metal.

We are interested in the flux of photons emitted into the far field, which we obtain from the Poynting vector. The latter can be calculated as $\mathbf{S} = \left(\frac{c}{4\pi}\right)\left(\mathbf{E}^{\mathrm{FF}} + \mathrm{c.c.}\right) \times \left(\mathbf{H}^{\mathrm{FF}} + \mathrm{c.c.}\right)$, in which the magnetic field is obtained from Faraday's law, $\mathbf{H}^{\mathrm{FF}} = -\left(\frac{\mathrm{i}}{k}\right) \nabla \times \mathbf{E}^{\mathrm{FF}}$. The time-averaged power flux is given by

$$\langle \hat{\boldsymbol{r}} \cdot \boldsymbol{S} \rangle_{time} = \frac{c}{2\pi r^2} |\boldsymbol{f}(\hat{r}, z_0)|^2,$$

where $\hat{\boldsymbol{r}} = \frac{\boldsymbol{k}}{k}$ is the unit vector indicating the emission direction. From here, we can write the flux of photons emitted per unit of solid angle as

$$\frac{d\phi(\theta, z_0)}{d\Omega} = \frac{r^2}{\hbar\omega}\langle\hat{\boldsymbol{r}} \cdot \boldsymbol{S}\rangle_{time} = \frac{c}{2\pi\hbar\omega}|\boldsymbol{f}(\hat{\boldsymbol{r}}, z_0)|^2$$

$$= \frac{1}{2\pi\hbar k}k^4p^2\left\{\left|\frac{k_{\rm mz}}{k_{\rm m}}\right|^2 D_{\rm p}^-(\theta, z_0) + D_{\rm s}^+(\theta, z_0) + \left|\frac{Q}{k_{\rm m}}\right|^2 D_{\rm p}^+(\theta, z_0)\right\}.$$

Integrating over emission solid angle (including azimuthal directions), we obtain the photoluminescence intensity emanating from a given induced dipole position at depth z_0 as

$$\phi(z_0) = \frac{k^3p(z_0)^2}{\hbar}\int_0^{\theta_{\max}} d\theta \left\{\left|\frac{k_{\rm mz}}{k_{\rm m}}\right|^2 D_{\rm p}^-(\theta, z_0) + D_{\rm s}^+(\theta, z_0) + \left|\frac{Q}{k_{\rm m}}\right|^2 D_{\rm p}^+(\theta, z_0)\right\}\sin(\theta), \qquad \text{(S2)}$$

where the integral extends over the angles allowed by the numerical aperture determined by the acceptance angle $\theta_{\max}$. Finally, we define the dipole strength per unit volume as $p^2 = \tilde{p}(z_0)^2 dV$ (with a specific dependence on z_0), allowing us to write the photon flux per unit area as

$$\tilde{\phi}(z_0) = \frac{k^3\tilde{p}(z_0)^2 dz_0}{\hbar}\int_0^{\theta_{\max}} d\theta \left\{\left|\frac{k_{\rm mz}}{k_{\rm m}}\right|^2 D_{\rm p}^-(\theta, z_0) + D_{\rm s}^+(\theta, z_0) + \left|\frac{Q}{k_{\rm m}}\right|^2 D_{\rm p}^+(\theta, z_0)\right\}\sin(\theta).$$

2. The role of electron transport

The total photon emission is then obtained by accumulating the incoherent sum of the emission produced by dipoles distributed along positions z_0 across the depth of the film. At each position inside the metal film, the emission is proportional to the dipole strength per unit volume $\tilde{p}(z_0)^2$, which is in turn dependent on incidence and emission photon energies. The dipole strength depends on the number of excited charges and their transport to the positions at which they produce luminescence, away from the locations where the excitation takes place. The actual mechanisms of transport are complex and follow an intricate dynamics in which a cascade of electron-hole pairs is generated, assisted by electron-electron interactions, as well as diffusion and scattering by phonons and impurities. Transport and dynamics are thus intimately related, as captured by the general expression

$$\tilde{p}(z_0) = \int_0^d dz_0' \; R(z_0, z_0', \omega_{\rm in}, \omega_{\rm out}) \, |\mathbf{E}^{\rm exc}(z_0', \omega_{\rm in})|^2,$$

which reflects the fact that the primary excitation at a location z_0' is proportional to the near-field intensity $|\mathbf{E}^{\rm exc}(z_0', \omega_{\rm in})|^2$ associated with the incident light (including its scattering by the planar interfaces; see Figure S5 and also an explicit expression below), as well as a nonlocal dynamics function $R(z_0, z_0', \omega_{\rm in}, \omega_{\rm out})$ (we denote the outgoing photon frequency as $\omega = \omega_{\rm out}$ for brevity in the above derivation). In what follows, we show that transport plays a negligible role by considering two limiting cases:

(1) *Local PL model*. In this limit, we consider that the excitation and emission processes take place at the same position, such that transport can be neglected. The emission dipole strength is then given, without loss of generality, by

$$\tilde{p}(z_0) = G(\omega_{\rm in}, \omega_{\rm out})\, |\mathbf{E}^{\rm exc}(z_0, \omega_{\rm in})|^2, \qquad \text{(S3a)}$$

which is just proportional to the near-field intensity $|\mathbf{E}^{\rm exc}(z_0, \omega_{\rm in})|^2$ at the emission position z_0. Here, the dynamics function $R(z_0, z_0', \omega_{\rm in}, \omega_{\rm out})$ is simplified under the local approximation (i.e., only parameters $z_0 = z_0'$ contribute), and further neglecting interface effects in the energy conversion process. We assume that $\tilde{p}^2(z_0)$ is linearly dependent on a position-independent conversion function $G(\omega_{\rm in}, \omega_{\rm out})$ that describes the local electron dynamics and the radiative recombination processes. In particular, $G(\omega_{\rm in}, \omega_{\rm out})$ can be computed just by considering the properties of the bulk metal, as obtained from the *ab-initio* calculations that we discuss below.

(2) *Maximally delocalized PL model*. In the opposite limit, any excitation inside the metal contributes to generate an emitting dipole at all film positions, such that

$$\tilde{p}^2 = G(\omega_{\rm in}, \omega_{\rm out}) \frac{1}{d} \int_0^d dz_0' \, |\mathbf{E}^{\rm exc}(z_0', \omega_{\rm in})|^2 \qquad \text{(S3b)}$$

becomes position-independent. Here, $G(\omega_{in}, \omega_{out})$ is the same function as in model (1).

We show in the main text that model (1) (local PL) agrees excellently with our measurements, whereas model (2) produces strong discrepancies.

3. Position integrated photoluminescence

In both transport models described by Eqs. (S3a) and (S3b), we calculate the photon flux per unit area by integrating the signal across the film as

$$\widetilde{PL}_{\rm external} = \int_0^d \tilde{\phi}(z_0).$$

Combining this expression with Eqs. (S2) and (S3), we find

$$\widetilde{PL}_{\rm external} = \frac{k^3}{\hbar} G(\omega_{\rm in}, \omega_{\rm out}) \int_0^d dz_0 \, |\mathbf{E}^{\rm exc}(z_0, \omega_{\rm in})|^2 \int_0^{\theta_{\rm max}} d\theta \, \left\{ \left|\frac{k_{\rm mz}}{k_{\rm m}}\right|^2 D_{\rm p}^{-}(\theta, z_0) + D_{\rm s}^{+}(\theta, z_0) + \left|\frac{Q}{k_{\rm m}}\right|^2 D_{\rm p}^{+}(\theta, z_0) \right\} \sin(\theta) \qquad \text{(S4a)}$$

in the local PL model, and

$$\widetilde{PL}_{\rm external} = \frac{k^3}{d\hbar} G(\omega_{\rm in}, \omega_{\rm out}) \left(\int_0^d dz_0' \, |\mathbf{E}^{\rm exc}(z_0', \omega_{\rm in})|^2 \right) \int_0^d dz_0 \int_0^{\theta_{\rm max}} d\theta \, \left\{ \left|\frac{k_{\rm mz}}{k_{\rm m}}\right|^2 D_{\rm p}^{-}(\theta, z_0) + D_{\rm s}^{+}(\theta, z_0) + \left|\frac{Q}{k_{\rm m}}\right|^2 D_{\rm p}^{+}(\theta, z_0) \right\} \sin(\theta) \qquad \text{(S4b)}$$

in the maximally delocalized PL model. These expressions can be related to the absorption power density (power per unit volume) inside the metal film by using the well-known relation [6]

$$P(z_0) = \frac{\omega_{\rm in}}{2\pi} \mathrm{Im}\{\epsilon_m(\omega_{\rm in})\} \, |\mathbf{E}^{\rm exc}(z_0, \omega_{\rm in})|^2.$$

In particular, Eqs. (S4a) and (S4b) become

$$\widetilde{PL}_{\rm external} = \frac{2\pi k^3}{\hbar\omega_{\rm in}\mathrm{Im}\{\epsilon_m(\omega_{\rm in})\}} G(\omega_{\rm in}, \omega_{\rm out}) \int_0^d dz_0 \; P(z_0) \int_0^{\theta_{\max}} d\theta \, \left\{ \left|\frac{k_{\rm mz}}{k_{\rm m}}\right|^2 D_{\rm p}^-(\theta, z_0) + D_{\rm s}^+(\theta, z_0) + \left|\frac{Q}{k_{\rm m}}\right|^2 D_{\rm p}^+(\theta, z_0) \right\} \sin(\theta) \quad \text{(S5a)}$$

and

$$\widetilde{PL}_{\rm external} = \frac{2\pi k^3}{\hbar\omega_{\rm in}\mathrm{Im}\{\epsilon_{\rm m}(\omega_{\rm in})\}} G(\omega_{\rm in}, \omega_{\rm out}) \frac{P}{d} \int_0^d dz_0 \int_0^{\theta_{\max}} d\theta \, \left\{ \left|\frac{k_{\rm mz}}{k_{\rm m}}\right|^2 D_{\rm p}^-(\theta, z_0) + D_{\rm s}^+(\theta, z_0) + \left|\frac{Q}{k_{\rm m}}\right|^2 D_{\rm p}^+(\theta, z_0) \right\} \sin(\theta), \quad \text{(S5b)}$$

respectively, where $P = \int_0^d dz_0 \; P(z_0)$ is the total absorbed power per unit of surface area.

4. Near-field excitation field intensity

For a plane wave normally impinging the metal surface (from medium ϵ_1) at wavelength $\lambda_{\rm in} = 2\pi c/\omega_{\rm in}$ with incident electric-field amplitude E_{in}, the electric field inside the slab is

$$\mathbf{E}_\sigma^{\rm exc}(z_0, \omega_{\rm in}) = E_{\rm in} \, \frac{t_{\sigma 1 \rm m}^{(\rm in)}}{1 - r_{\sigma \rm m1}^{(\rm in)} r_{\sigma \rm m3}^{(\rm in)} e^{2{\rm i}k_{\rm mz}^{(\rm in)} d}} \left[\mathbf{e}_\sigma^- \, {\rm e}^{-{\rm i}k_{\rm mz}^{(\rm in)}(d - z_0)} + \mathbf{e}_\sigma^+ r_{\sigma \rm m3}^{(\rm in)} {\rm e}^{{\rm i}k_{\rm mz}^{(\rm in)}(d + z_0)} \right] = E_{\rm in} C(z_0),$$

where $k_{\rm mz}^{(\rm in)} = 2\pi/\lambda_{\rm in}\sqrt{\epsilon_{\rm m}(\lambda_{\rm in})}$, while $\sigma = \{\rm s, p\}$ indicates the light polarization. In particular, under the conditions of the present experiment, we can approximate the light incidence to be normal to the film, and then, we have $r_{\rm p}^{(\rm in)} = -r_{\rm s}^{(\rm in)}$ and $t_{\rm p}^{(\rm in)} = t_{\rm s}^{(\rm in)}$, so that the intensity is independent of the incident light polarization.

5. Connection with DFT calculations: equating dipole strength with internal photoluminescence

For ease of notation, we make a connection between the emission dipole strength p^2 and direct optical transitions, noting that the same analysis can be carried out for phonon-assisted and pre-scattered

transitions and we obtain analogous prefactors. Within the DFT formalism, we obtain the total density of dipole strength (normalized per unit volume and emission frequency $\omega = \omega_{\text{out}}$) due to direct electron-hole-recombination transitions as

$$\frac{p^2_{\text{direct}}}{dV d\omega_{\text{out}}} = \frac{\hbar e^2}{m_e^2 \omega_{\text{out}}^2} \int_{BZ} \frac{g_s d\vec{k}}{(2\pi)^3} \sum_{n',n} f_{\vec{k},n'} \left(1 - f_{\vec{k},n}\right) \delta\left(\varepsilon_{\vec{k}n'} - \varepsilon_{\vec{k}n} - \hbar\omega_{\text{out}}\right) \left|\vec{p}^{\vec{k}}_{n,n'}\right|^2.$$

Here, e is the charge of an electron, m_e is the mass of an electron, $g_s = 2$ is the spin degeneracy, $f_{\vec{k},n}$ is the probability that a one-electron state with wave vector $\vec{k}$ and band index n is occupied (i.e., the occupation factor), $\varepsilon_{\vec{k}n}$ the energy eigenvalue of the relevant state, $\hbar$ Planck's constant divided by 2π and $\vec{p}^{\vec{k}}_{n,n'}$ matrix elements of the momentum operator. See Supplemental Note 8 and Brown *et al.* [7] for more details. Furthermore, we can write the spontaneous emission rate of photons per unit ω_{out} and volume from an isotropic/cubic crystal (see Supplemental Note 8 for further details) as [8]

$$\Gamma_{\text{direct}}(\omega_{\text{in}}, \omega_{\text{out}}) = \frac{4e^2 \omega_{\text{out}} \text{Re}\{\sqrt{\epsilon_{\text{m}}(\omega_{\text{out}})}\}}{m_e^2 c^3} \int_{BZ} \frac{g_s d\vec{k}}{(2\pi)^3} \sum_{n',n} f_{\vec{k},n'} \left(1 - f_{\vec{k},n}\right) \delta\left(\varepsilon_{\vec{k}n'} - \varepsilon_{\vec{k}n} - \hbar\omega_{\text{out}}\right) \left|\vec{p}^{\vec{k}}_{n,n'}\right|^2$$

and therefore

$$\frac{\tilde{p}^2_{\text{direct}}}{d\omega_{\text{out}}} = \frac{\hbar}{4k^3 \text{Re}\left\{\sqrt{\epsilon_{\text{m}}(\omega_{\text{out}})}\right\}} \Gamma_{\text{direct}}(\omega_{\text{in}}, \omega_{\text{out}}, z_0). \qquad (S6)$$

The same prefactors are obtained for phonon-assisted or pre-scattered luminescence. Here we are specifically interested in emission beyond that of a black body at equilibrium. As discussed in Supplemental Note 8, in this case we can derive a spontaneous emission rate per absorbed photon, $PL_{\text{internal}}(\omega_{\text{in}}, \omega_{\text{out}})$ for the luminescence due to laser excitation, and the number of locally absorbed photons per unit volume is $\frac{P(z_0)}{\hbar\omega_{\text{in}}}$. Thin film effects are accounted for in the above dipole formalism, so we consider the bulk $PL_{\text{internal}}(\omega_{\text{in}}, \omega_{\text{out}})$ in a similar way as in the context of semiconductor PL, although we caution that the physical meaning of internal photon emission is compromised by losses and the deep-subwaverlength thickness of the films in the present work. In the case of minimal charge redistribution prior to luminescence we can state that the local laser-stimulated spontaneous emission rate is

$$\Gamma = PL_{\text{internal}}(\omega_{\text{in}}, \omega_{\text{out}}) \frac{P(z_0)}{\hbar\omega_{\text{in}}}$$

while in the maximally delocalized PL model the laser-stimulated spontaneous emission rate

$$\Gamma = PL_{\text{internal}}(\omega_{\text{in}}, \omega_{\text{out}}) \frac{1}{d} \int \frac{P(z_0{}')}{\hbar\omega_{\text{in}}} dz_0'.$$

Taking equations S3a and S6 allows us to equate

$$\begin{aligned} \tilde{p}_{\text{direct}}^2(z_0) &= G(\omega_{\text{in}}, \omega_{\text{out}})\, |\mathbf{E}^{\text{exc}}(z_0, \omega_{\text{in}})|^2 \\ &= \frac{\hbar}{4k^3 \text{Re}\left\{\sqrt{\epsilon_{\text{m}}(\omega_{\text{out}})}\right\}} PL_{\text{internal}}(\omega_{\text{in}}, \omega_{\text{out}}) \frac{P(z_0)}{\hbar\omega_{\text{in}}} d\omega_{\text{out}} \end{aligned}$$

and from equation S3b

$$\begin{aligned} \tilde{p}_{\text{direct}}^2 &= G(\omega_{\text{in}}, \omega_{\text{out}}) \frac{1}{d} \int_0^d dz_0' \; |\mathbf{E}^{\text{exc}}(z_0', \omega_{\text{in}})|^2 \\ &= \frac{\hbar}{4k^3 \text{Re}\left\{\sqrt{\epsilon_{\text{m}}(\omega_{\text{out}})}\right\}} PL_{\text{internal}}(\omega_{\text{in}}, \omega_{\text{out}}) \frac{1}{d} \int \frac{P(z_0{}')}{\hbar\omega_{\text{in}}} dz_0' \, d\omega_{out}. \end{aligned}$$

In both cases this gives

$$G(\omega_{\text{in}}, \omega_{\text{out}}) = \frac{\hbar PL_{\text{internal}}(\omega_{\text{in}}, \omega_{\text{out}}) \frac{\omega_{\text{in}}}{2\pi} \text{Im}\{\epsilon_{\text{m}}(\omega_{\text{in}})\} d\omega_{\text{out}}}{4k^3 \text{Re}\left\{\sqrt{\epsilon_{\text{m}}(\omega_{\text{out}})}\right\} \hbar\omega_{\text{in}}}.$$

Substituting into Eqs. (S5a) and (S5b), and dividing by $d\omega_{\text{out}}$ to give the recorded signal per unit area, per unit ω_{out}, we obtain

$$\begin{aligned} PL_{\text{external}} = \frac{PL_{\text{internal}}}{4\text{Re}\left\{\sqrt{\epsilon_{\text{m}}(\omega_{\text{out}})}\right\}} \int_0^d dz_0 \left(\frac{P(z_0)}{\hbar\omega_{\text{in}}}\right) \int_0^{\theta_{max}} d\theta \, \Bigg\{ \left|\frac{k_{\text{mz}}}{k_{\text{m}}}\right|^2 D_{\text{p}}^-(\theta, z_0) + D_{\text{s}}^+(\theta, z_0) \\ + \left|\frac{Q}{k_{\text{m}}}\right|^2 D_{\text{p}}^+(\theta, z_0) \Bigg\} \sin(\theta) \end{aligned}$$

for the local PL model and

$$PL_{\text{external}} = \frac{PL_{\text{internal}}}{4d\mathrm{Re}\left\{\sqrt{\epsilon_{\text{m}}(\omega_{\text{out}})}\right\}}\left(\frac{P}{\hbar\omega_{\text{in}}}\right)\int_0^d dz_0 \int_0^{\theta_{max}} d\theta \left\{\left|\frac{k_{\text{mz}}}{k_{\text{m}}}\right|^2 D_{\text{p}}^{-}(\theta,z_0) + D_{\text{s}}^{+}(\theta,z_0) + \left|\frac{Q}{k_{\text{m}}}\right|^2 D_{\text{p}}^{+}(\theta,z_0)\right\}\sin(\theta)$$

for the maximally delocalised model. We note the factor of 4 in the denominator originates from the definition of PL_{internal} (in accordance with the semiconductor luminescence community) rather than the electromagnetic derivation.

6. Simplifying notation

We can re-write

$$P(z_0) = \frac{\omega_{\text{in}}}{2\pi}\mathrm{Im}\{\epsilon_{\text{m}}(\omega_{\text{in}})\}\,|\mathbf{E}^{\text{exc}}(z_0,\omega_{\text{in}})|^2 = \frac{\omega_{\text{in}}}{2\pi}\mathrm{Im}\{\epsilon_{\text{m}}(\omega_{\text{in}})\}|E_{\text{in}}|^2|C(z_0)|^2 = f_{\text{abs}}(z_0,\lambda_{\text{in}})I_{\text{in}},$$

where I_{in} is the incident intensity that can be experimentally recorded (via power meter and spot-size measurements) and f_{abs} describes the number of photons absorbed at z_0 (per unit length). We have changed from using ω to wavelength λ as experiments are recorded as a function of wavelength. We group all other terms, which describe energy loss prior to photons escaping the film, as

$$f_{\text{emit}}(z_0,\lambda_{\text{out}}) = \int_0^{\theta_{\max}} d\theta \left\{\left|\frac{k_{\text{mz}}}{k_{\text{m}}}\right|^2 D_{\text{p}}^{-} + D_{\text{s}}^{+} + \left|\frac{Q}{k_{\text{m}}}\right|^2 D_{\text{p}}^{+}\right\}\left(\frac{\sin(\theta)}{4Re\left\{\sqrt{\epsilon_{\text{m}}(\omega_{\text{out}})}\right\}}\right).$$

This allows us to write the two models as

$$PL_{\text{external}} = PL_{\text{internal}}\left(\frac{I_{\text{in}}}{\hbar\omega_{\text{in}}}\right)\int_0^d dz_0\, f_{\text{abs}}(z_0,\lambda_{\text{in}})\, f_{\text{emit}}(z_0,\lambda_{\text{out}})$$

for local PL and

$$PL_{\text{external}} = \frac{1}{\mathrm{d}}PL_{\text{internal}}(\omega_{\text{in}},\omega_{\text{out}})\left(\frac{I_{\text{in}}}{\hbar\omega_{\text{in}}}\right)\int_0^d dz_0'\; f_{\text{abs}}(z_0',\lambda_{\text{in}})\int_0^d dz_0\; f_{\text{emit}}(z_0,\lambda_{\text{out}})$$

for maximally delocalised PL. Finally, as an example, in Figure S6 we plot $\int dz_0 f_{\text{abs}}(z_0,\lambda_{\text{in}}) f_{\text{emit}}(z_0,\lambda_{\text{out}})$ for an 88 nm flake with 488 nm excitation and NA=0.7.

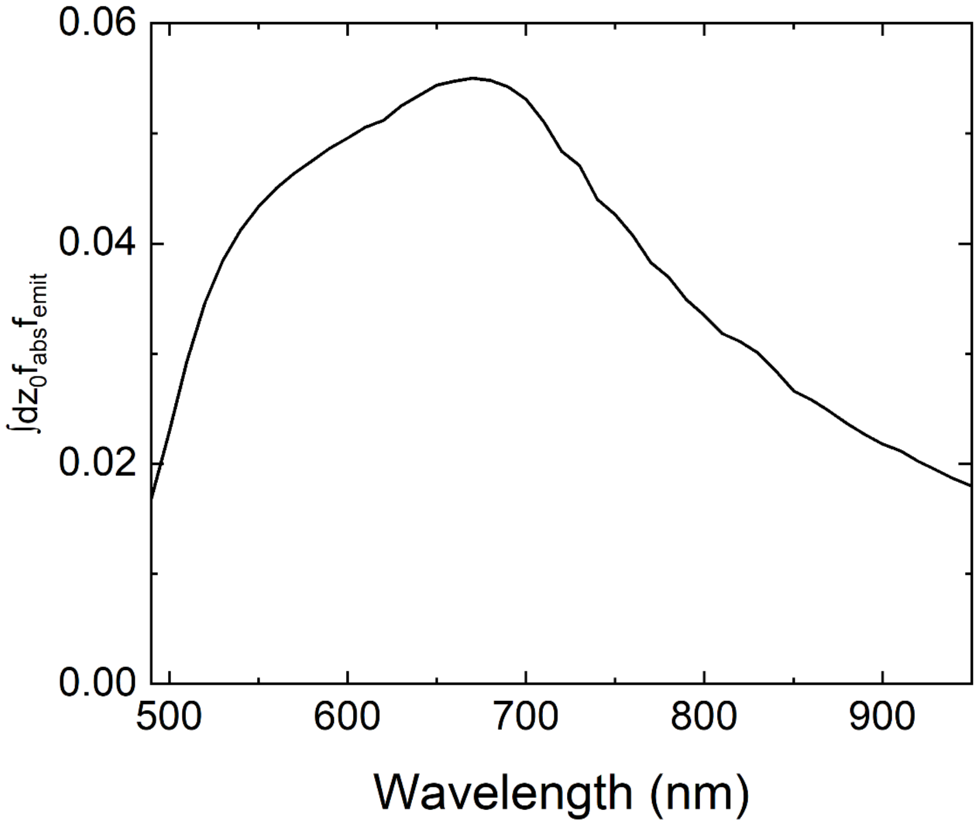

Figure S6. $\int dz_0 f_{\mathrm{abs}}(z_0, \lambda_{\mathrm{in}}) f_{\mathrm{emit}}(z_0, \lambda_{\mathrm{out}})$ *as a function of wavelength for an 88 nm flake when exciting at 488 nm and observing with a microscope objective NA=0.7.*

Supplemental Note 7 – Measuring flake absorption with sample thickness

1. <u>Light absorption model</u>

We consider gold on top of glass (as per our measurements), so

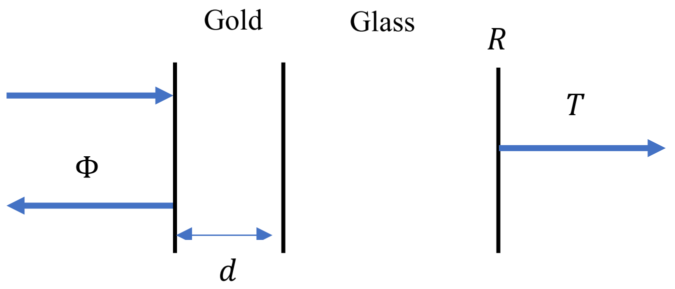

We consider that there is interference in gold, but all coherence is lost while light travels through the glass layer. Furthermore, we only write the formula for s polarization as, for light incident perpendicular on the sample, the two polarisations give the same result. If we consider gold has power transmission and reflection coefficients $R_{\mathrm{L}}, R_{\mathrm{R}}, T_{\mathrm{L}\rightarrow\mathrm{R}}$ and $T_{\mathrm{R}\rightarrow\mathrm{L}}$ (where L and R correspond to the left and right sides of gold), we can derive

$$T = \frac{(1-R)T_{\mathrm{L}\rightarrow\mathrm{R}}}{1-R_{\mathrm{R}}R} \text{ and } \Phi = R_{\mathrm{L}} + \frac{T_{\mathrm{R}\rightarrow\mathrm{L}}T_{\mathrm{L}\rightarrow\mathrm{R}}R}{1-R_{\mathrm{R}}R}.$$

We can state

$$R_L = \left|\frac{-r_{\mathrm{s,m1}}\mathrm{e}^{-\mathrm{i}k_{\mathrm{m}}d} + r_{\mathrm{s,m3}}\mathrm{e}^{\mathrm{i}k_{\mathrm{m}}d}}{\mathrm{e}^{-\mathrm{i}k_{\mathrm{m}}d} - r_{\mathrm{s,m1}}r_{\mathrm{s,m3}}\mathrm{e}^{\mathrm{i}k_{\mathrm{m}}d}}\right|^2, R_R = \left|\frac{-r_{\mathrm{s,m3}}\mathrm{e}^{-\mathrm{i}k_{\mathrm{m}}d} + r_{\mathrm{s,m1}}\mathrm{e}^{\mathrm{i}k_{\mathrm{m}}d}}{\mathrm{e}^{-\mathrm{i}k_{\mathrm{m}}d} - r_{\mathrm{s,m1}}r_{\mathrm{s,m3}}\mathrm{e}^{\mathrm{i}k_{\mathrm{m}}d}}\right|^2, T_{L\rightarrow R} = \frac{n_3}{n_1}\left|\frac{t_{\mathrm{s,1m}}t_{s,\mathrm{m3}}}{\mathrm{e}^{-\mathrm{i}k_{\mathrm{m}}d} - r_{\mathrm{s,m1}}r_{\mathrm{s,m3}}\mathrm{e}^{\mathrm{i}k_{\mathrm{m}}d}},\right|^2 \text{ and } T_{R\rightarrow L} = \frac{n_1}{n_3}\left|\frac{t_{\mathrm{s,m1}}t_{s,\mathrm{3m}}}{\mathrm{e}^{-\mathrm{i}k_{\mathrm{m}}d} - r_{\mathrm{s,m1}}r_{\mathrm{s,m3}}\mathrm{e}^{\mathrm{i}k_{\mathrm{m}}d}}\right|^2$$

where n_3 is the refractive index of quartz. We can write for normal incidence

$$t_{\mathrm{s,1m}} = \frac{2n_1}{n_1 + n_{\mathrm{m}}}, t_{\mathrm{s,m3}} = \frac{2n_{\mathrm{m}}}{n_{\mathrm{m}} + n_3}, t_{\mathrm{s,3m}} = \frac{2n_3}{n_{\mathrm{m}} + n_3}$$

and

$$R = \left|\frac{n_1 - n_3}{n_1 + n_3}\right|^2.$$

Assuming that there is no absorption in the quartz, we can say the total absorption in the gold is

$$Abs = 1 - T - \Phi.$$

We note that this gives extremely similar values of absorption to the cases modelled with no substrate.

2. Experimental results

We present the reflection and transmission of a thin (14 nm) and moderately thick (47 nm) gold flake in Figures S7a and b respectively. In both cases we fit this data with the model presented in the previous section using McPeak's optical constants [9] (noting that other optical constants give similar results). The only free parameter in this fitting is the thickness of the flake. We present a comparison of this fitted thicknesses with that measured via atomic force microscopy (AFM) in Figure S7c, which shows strong agreement. This demonstrates that for both thin and thick flakes, optical constants in the literature are sufficient to understand the absorption properties of these flakes. We note that no additional factors were needed to model thin flakes, in agreement with Großmann et al. [10].

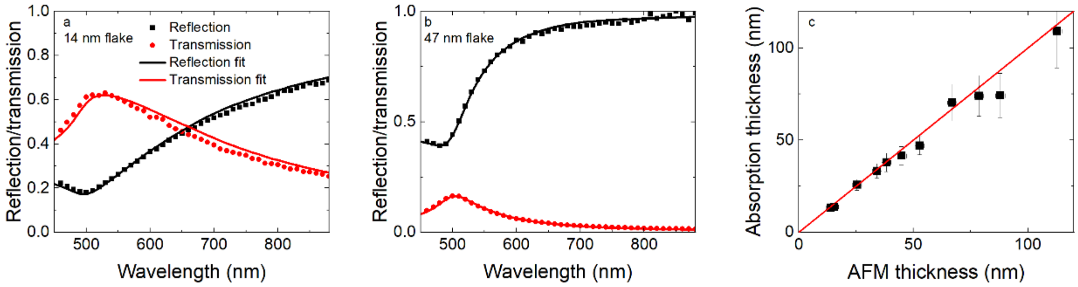

Figure S7. a) and b) present data and fits of sample reflection and transmission for 14 nm and 47 nm flake respectively. c) Thicknesses extracted from absorption data relative to those measured from atomic force microscopy (AFM), with the red line showing the expected agreement. The legend in a) also applies to b).

Supplemental Note 8 – Modelling PL from DFT

1. Post-scattered carriers

In first-principles calculations, the imaginary part of the dielectric function for direct and phonon-assisted transitions is calculated using (we denote the outgoing photon frequency as $\omega = \omega_{out}$ for brevity in the following derivation):

$$\mathrm{Im}\left(\epsilon_{\mathrm{m,direct}}(\omega)\right) = \frac{4\pi^2 e^2}{m_{\mathrm{e}}^2\omega^2}\int_{BZ}\frac{g_s d\vec{k}}{(2\pi)^3}\sum_{n',n}(f_{\vec{k},n} - f_{\vec{k},n'})\delta(\varepsilon_{\vec{k}n'} - \varepsilon_{\vec{k}n} - \hbar\omega)\left|\vec{p}^{\vec{k}}_{n,n'}\right|^2.$$

$$\mathrm{Im}\left(\epsilon_{\mathrm{m,phonon}}(\omega)\right) = \frac{4\pi^2 e^2}{m_{\mathrm{e}}^2\omega^2}\int_{BZ}\frac{g_s d\vec{k}' d\vec{k}}{(2\pi)^6}\sum_{n'n\alpha\pm}\left(f_{\vec{k}n} - f_{\vec{k}'n'}\right)\left(n_{\vec{k}'-\vec{k},\alpha} + \frac{1}{2} \mp \frac{1}{2}\right)\delta\left(\varepsilon_{\vec{k}'n'} - \varepsilon_{\vec{k}n} - \hbar\omega \mp \hbar\omega_{\vec{k}'-\vec{k},\alpha}\right)\left|\sum_{n_1}\left(\frac{g^{\vec{k}'-\vec{k},\alpha}_{\vec{k}'n',\vec{k}n_1}\vec{p}^{\vec{k}}_{n_1 n}}{\varepsilon_{\vec{k}n_1} - \varepsilon_{\vec{k}n} - \hbar\omega + i\eta} + \frac{\vec{p}^{\vec{k}'}_{n'n_1} g^{\vec{k}'-\vec{k},\alpha}_{\vec{k}'n_1,\vec{k}n}}{\varepsilon_{\vec{k}'n_1} - \varepsilon_{\vec{k}n} \mp \hbar\omega_{\vec{k}'-\vec{k},\alpha} + i\eta}\right)\right|^2$$

Here e is the charge of an electron, m_{e} the mass of an electron, g_s is the spin degeneracy factor, $f_{\vec{k},n}$ the probability a state with wavevector $\vec{k}$ and band index n is occupied (the occupation factor), $\varepsilon_{\vec{k}n}$ the energy eigenvalue of the relevant state, $\hbar$ Planck's constant divided by 2π and $\vec{p}^{\vec{k}}_{n,n'}$ matrix elements of the momentum operator, $n_{\vec{k}'-\vec{k},\alpha}$ the phonon occupation of the relevant state, $g^{\vec{k}'-\vec{k},\alpha}_{\vec{k}'n',\vec{k}n_1}$ the electron-phonon matrix elements with phonon mode index α and η a small value to aid with calculation. See Brown et al. for more discussion of defined terms [7]. This can be further histogrammed by ϵ to compute hot carrier distributions, as implemented in JDFTx [11].

We present a derivation of PL for the case of direct transitions, and present the final equation for direct and phonon assisted transitions. We can write the spontaneous emission rate of photons per unit ω and volume from an isotropic/cubic crystal as [8]

$$\Gamma(\omega) = \frac{4e^2\omega}{m_{\mathrm{e}}^2 c^3}\mathrm{Re}\{\sqrt{\epsilon_{\mathrm{m}}(\omega)}\}\int_{BZ}\frac{g_s d\vec{k}}{(2\pi)^3}\sum_{n',n} f_{\vec{k},n'}(1 - f_{\vec{k},n})\delta(\varepsilon_{\vec{k}n'} - \varepsilon_{\vec{k}n} - \hbar\omega)\left|\vec{p}^{\vec{k}}_{n,n'}\right|^2 \quad \text{(S7)}$$

where $\mathrm{Re}\{\sqrt{\epsilon_{\mathrm{m}}(\omega)}\}$ is the factor from the local photonic density of states [12]. To calculate luminescence from a perturbed electron distribution, let $f_{\vec{k},n} = f_0(\epsilon_{\vec{k}n}) + \delta f(\epsilon_{\vec{k}n}) \approx \Theta(\mu - \epsilon_{\vec{k}n}) +$

$\delta f(\epsilon_{\vec{k}n})$, where Θ is a step function, μ the Fermi level f_0 is the Fermi distribution and δf is a small perturbation which we approximate in our dynamics code as only depending on the energy (i.e. assuming momentum information is rapidly lost or irrelevant). This is justified in the case of weak laser excitation, as was the case in all our experiments. We can write the change of emission relative to equilibrium (i.e. difference from the black-body spectrum) as:

$$\delta\Gamma_{\text{scatt,direct}}(\omega) = \int d\varepsilon \delta f(\varepsilon)\Gamma_{\text{direct}}(\varepsilon,\omega),$$

where

$$\Gamma_{\text{direct}}(\varepsilon,\omega) = \frac{4e^2\omega}{m_e^2c^3}\text{Re}\{\sqrt{\epsilon_{\text{m}}(\omega)}\}\int_{BZ}\frac{g_s d\vec{k}}{(2\pi)^3}\sum_{n',n}\left(\delta(\varepsilon-\varepsilon_{\vec{k}n'})f_{\vec{k},n}-f_{\vec{k},n'}\delta(\varepsilon-\varepsilon_{\vec{k}n})\right)\delta(\varepsilon_{\vec{k}n'} - \varepsilon_{\vec{k}n}-\hbar\omega)\left|\vec{p}^{\vec{k}}_{\alpha,n,n'}\right|^2,$$

for direct transitions and

$$\delta\Gamma_{\text{scatt,phonon}}(\omega) = \int d\varepsilon \delta f(\varepsilon)\Gamma_{\text{phonon}}(\varepsilon,\omega),$$

with

$$\Gamma_{\text{phonon}}(\varepsilon,\omega) = \frac{4e^2\omega}{m_{\text{e}}^2c^3}\text{Re}\{\sqrt{\epsilon_{\text{m}}(\omega)}\}\int_{BZ}\frac{g_s d\vec{k}'d\vec{k}}{(2\pi)^6}\sum_{n'n\alpha\pm}\left(\delta(\varepsilon-\varepsilon_{\vec{k}n'})f_{\vec{k},n} - f_{\vec{k},n'}\delta(\varepsilon-\varepsilon_{\vec{k}n})\right)\left(n_{\vec{k}'-\vec{k},\alpha}+\frac{1}{2}\mp\frac{1}{2}\right)\delta(\varepsilon_{\vec{k}'n'}-\varepsilon_{\vec{k}n}-\hbar\omega \mp \hbar\omega_{\vec{k}'-\vec{k},\alpha})\left|\sum_{n_1}\left(\frac{g^{\vec{k}'-\vec{k},\alpha}_{\vec{k}'n',\vec{k}n_1}\vec{p}^{\vec{k}}_{n_1n}}{\varepsilon_{\vec{k}n_1}-\varepsilon_{\vec{k}n}-\hbar\omega+i\eta}+\frac{\vec{p}^{\vec{k}'}_{n'n_1}g^{\vec{k}'-\vec{k},\alpha}_{\vec{k}'n_1,\vec{k}n}}{\varepsilon_{\vec{k}'n_1}-\varepsilon_{\vec{k}n}\mp\hbar\omega_{\vec{k}'-\vec{k},\alpha}+i\eta}\right)\right|^2$$

for phonons. The total post-scattered luminescence can therefore be written as

$$\delta\Gamma_{\text{scatt}}(\omega) = \int d\varepsilon\delta f(\varepsilon)(\Gamma_{\text{direct}}(\varepsilon,\omega)+\Gamma_{\text{phonon}}(\varepsilon,\omega)).$$

We note that for both direct and phonon assisted transitions in $\left(\delta(\varepsilon-\varepsilon_{\vec{k}n'})f_{\vec{k},n}-f_{\vec{k},n'}\delta(\varepsilon-\varepsilon_{\vec{k}n})\right)$ the first term corresponds to unexcited electrons and the second to unexcited holes. Consequently, we can define a quantity just like the carrier distribution output which contains the carrier-resolved emission contribution. We present $\Gamma_{\text{direct}}(\varepsilon,\omega)+\Gamma_{\text{phonon}}(\varepsilon,\omega)$ in Figure S8. After we evaluate this

once, we can integrate against δf for a specific perturbed case to evaluate its emission. δf is discussed further in section 3.

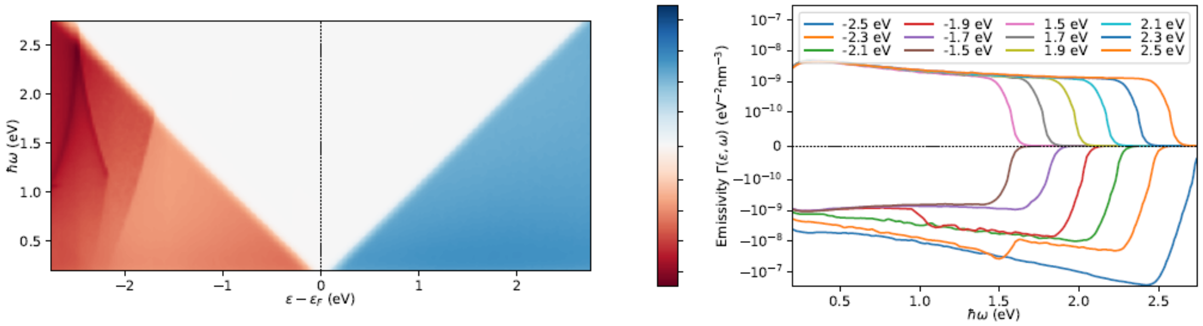

Figure S8. Emissivity per unit volume, $\Gamma_{\text{direct}}(\varepsilon,\omega)+\Gamma_{\text{phonon}}(\varepsilon,\omega)$, as a function of emission energy ($\hbar\omega$) and excited electron/hole energy ($\epsilon-\epsilon_F$). Plot on right presents lineslices of main plot at different electron/hole energies, as marked on legend.

2. <u>Pre-scattered carriers</u>

The above approximation of working with δf may be inaccurate for emission from excited carriers prior to any scattering. We can account for this by estimating the emission rate from the unscattered carriers prior to scattering by getting back to the same initial state. This would emit a photon with the same nominal frequency as the absorption (except for broadening). However, due to short hole lifetimes the broadening effect is significant. We can therefore collect contributions corresponding to unscattered carriers at energy ε due to absorption and emission of photons both at frequency ω.

Starting from the expression for $\mathrm{Im}(\epsilon_{\mathrm{m}}(\omega))$ at the start of this note, the rate of change in electron and hole occupations due to absorption rate $a(\omega_{\mathrm{in}})$ of a photons of frequency ω_{in} per unit volume is (written with the n and n' contributions, that is filled and empty energy levels, separately for simplicity using accumulation operators):

$$\dot{f}_{\vec{k},n} -= \frac{a(\omega_{\mathrm{in}})}{\mathrm{Im}(\epsilon_{\mathrm{m}}(\omega_{\mathrm{in}}))}\cdot\frac{4\pi^2 e^2}{m_{\mathrm{e}}^2\omega_{\mathrm{in}}{}^2}\sum_{n'}(f_{\vec{k},n}-f_{\vec{k},n'})\delta(\varepsilon_{\vec{k}n'}-\varepsilon_{\vec{k}n}-\hbar\omega_{\mathrm{in}})\left|\vec{p}^{\vec{k}}_{\alpha,n',n}\right|^2$$

$$\dot{f}_{\vec{k},n'} += \frac{a(\omega_{\mathrm{in}})}{\mathrm{Im}(\epsilon_{\mathrm{m}}(\omega_{\mathrm{in}}))}\cdot\frac{4\pi^2 e^2}{m_{\mathrm{e}}^2\omega_{\mathrm{in}}{}^2}\sum_{n'}(f_{\vec{k},n}-f_{\vec{k},n'})\delta(\varepsilon_{\vec{k}n'}-\varepsilon_{\vec{k}n}-\hbar\omega_{\mathrm{in}})\left|\vec{p}^{\vec{k}}_{\alpha,n',n}\right|^2.$$

With carrier lifetimes $\tau_{\vec{k},n}$, this results in a steady-state change of occupation given by:

$$\delta\dot{f}_{\vec{k},n} -= \tau_{\vec{k},n}\frac{a(\omega_{\mathrm{in}})}{\mathrm{Im}(\epsilon_{\mathrm{m}}(\omega_{\mathrm{in}}))}\cdot\frac{4\pi^2 e^2}{m_{e}^2\omega_{\mathrm{in}}{}^2}\sum_{n'}(f_{\vec{k},n}-f_{\vec{k},n'})\delta(\varepsilon_{\vec{k}n'}-\varepsilon_{\vec{k}n}-\hbar\omega_{\mathrm{in}})\left|\vec{p}^{\vec{k}}_{\alpha,n',n}\right|^2$$

$$\delta \dot{f}_{\vec{k},n'} += \tau_{\vec{k},n'} \frac{a(\omega_{\text{in}})}{\text{Im}\big(\epsilon_{\text{m}}(\omega_{\text{in}})\big)} \cdot \frac{4\pi^2 e^2}{m_e^2 {\omega_{\text{in}}}^2} \sum_{n'} (f_{\vec{k},n} - f_{\vec{k},n'}) \delta\big(\varepsilon_{\vec{k}n'} - \varepsilon_{\vec{k}n} - \hbar\omega_{\text{in}}\big) \left|\vec{p}^{\vec{k}}_{\alpha,n',n}\right|^2.$$

The corresponding change in emission rate of photons per unit volume and unit ω_{out} from the same channel (i.e. just reversing the above absorption) is:

$$\delta\Gamma_{\text{pre-scatt}}(\omega_{\text{out}}) = \frac{4e^2\omega_{\text{out}}}{m_{\text{e}}^2 c^3} \text{Re}\left\{\sqrt{\epsilon_{\text{m}}(\omega_{\text{out}})}\right\} \int_{BZ} \frac{g_{\text{s}} d\vec{k}}{(2\pi)^3} \sum_{n',n} \big(\delta f_{\vec{k},n'}(1 - f_{\vec{k},n}) - f_{\vec{k},n'} \delta f_{\vec{k},n}\big) \delta\big(\varepsilon_{\vec{k}n'} - \varepsilon_{\vec{k}n} - \hbar\omega_{\text{out}}\big) \left|\vec{p}^{\vec{k}}_{\alpha,n,n'}\right|^2.$$

Substituting in for $\delta f_{\vec{k},n'}$ and $\delta f_{\vec{k},n}$ we obtain

$$\delta\Gamma_{\text{pre-scatt}}(\omega_{\text{out}}) = \frac{a(\omega_{\text{in}})}{\text{Im}\big(\epsilon_{\text{m}}(\omega_{\text{in}})\big)} \cdot \frac{4\pi^2 e^2}{m_{\text{e}}^2 {\omega_{\text{in}}}^2} \int_{BZ} \frac{g_{\text{s}} d\vec{k}}{(2\pi)^3} \sum_{n',n} (f_{\vec{k},n} - f_{\vec{k},n'}) \delta\big(\varepsilon_{\vec{k}n'} - \varepsilon_{\vec{k}n} - \hbar\omega_{\text{in}}\big) \left|\vec{p}^{\vec{k}}_{\alpha,n',n}\right|^2$$
$$\times \frac{4e^2\omega_{\text{out}}}{m_{\text{e}}^2 c^3} \text{Re}\left\{\sqrt{\epsilon_{\text{m}}(\omega_{\text{out}})}\right\} \big(\tau_{\vec{k},n'}(1 - f_{\vec{k},n}) + f_{\vec{k},n'}\tau_{\vec{k},n}\big) \delta(\hbar\omega_{\text{in}} - \hbar\omega_{\text{out}}) \left|\vec{p}^{\vec{k}}_{\alpha,n',n}\right|^2.$$

As expected, the pre-scattered emission has to be at the same frequency (up to broadening due to $\tau_{\vec{k},n}$). We can therefore calculate the total self-emission ($\int d\omega_{\text{in}}$ of above) and add in the Lorentzian frequency dependence. Normalized per photon absorbed, the self-emission probability (dimensionless) is therefore:

$$\frac{\delta\Gamma_{\text{pre-scatt}}(\omega_{\text{out}})}{\text{a}(\omega_{\text{in}})} = \frac{1}{\text{Im}\big(\epsilon_{\text{m}}(\omega_{\text{in}})\big)} \frac{4\pi^2 e^2}{\hbar m_{\text{e}}^2 {\omega_{\text{in}}}^2} \int_{BZ} \frac{g_{\text{s}} d\vec{k}}{(2\pi)^3} \sum_{n',n} (f_{\vec{k},n} - f_{\vec{k},n'}) \delta\big(\varepsilon_{\vec{k}n'} - \varepsilon_{\vec{k}n} - \hbar\omega_{\text{in}}\big) \left|\vec{p}^{\vec{k}}_{\alpha,n',n}\right|^2$$
$$\times \frac{4e^2\omega_{\text{out}}}{m_{\text{e}}^2 c^3} \text{Re}\{\sqrt{\epsilon_{\text{m}}(\omega_{\text{out}})}\} \big(\tau_{\vec{k},n'}(1 - f_{\vec{k},n}) + f_{\vec{k},n'}\tau_{\vec{k},n}\big) \left|\vec{p}^{\vec{k}}_{\alpha,n',n}\right|^2 \frac{\gamma_{\vec{k}n'n}}{\pi\left[(\omega_{\text{in}} - \omega_{\text{out}})^2 + \gamma_{\vec{k}n',n}^2\right]},$$

where the Lorentzian broadening is based on the inverse carrier lifetimes $\gamma_{\vec{k}n'n} = \frac{\tau^{-1}_{\vec{k}n'} + \tau^{-1}_{\vec{k}n}}{2}$ due to electron-electron and electron-phonon scattering (this emerges from the imaginary part of the self energy, $\text{Im}\Sigma_{\vec{k}n} = \frac{\hbar\tau^{-1}_{\vec{k}n}}{2}$, of each carrier in the energy-conserving δ-function). Finally, we note that $PL_{\text{internal}} = \delta\Gamma_{\text{scatt}} + \delta\Gamma_{\text{pre-scatt}}$. δf is discussed further in section 3.

We do not account for phonon-assisted transitions in the self-emission as only direct transitions would be sensitive to k-point smearing. We present $\delta\Gamma_{\text{pre-scatt}}(\omega_{\text{out}})$ in Figure S9.

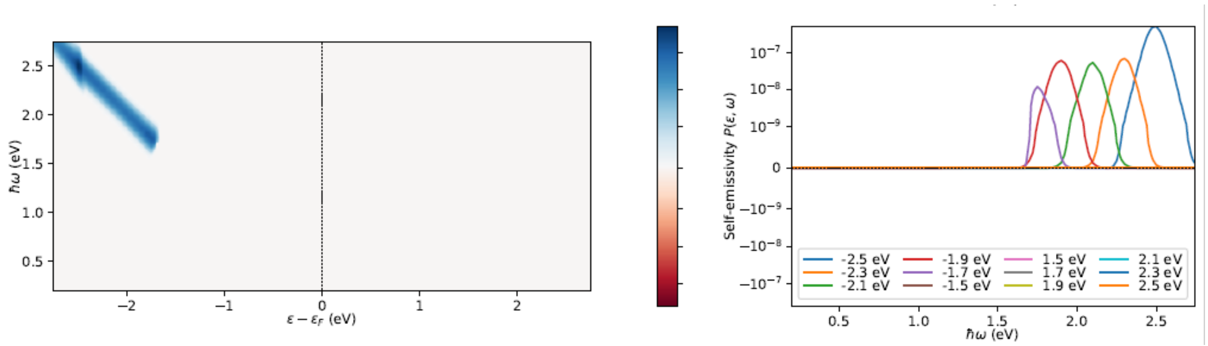

Figure S9. Self-emissivity per unit volume, $\delta\Gamma_{\text{pre-scatt}}(\omega_{\text{out}})$, as a function of emission energy ($\hbar\omega_{\text{out}}$) and excited electron/hole energy ($\epsilon - \epsilon_F$). Plot on right presents lineslices of main plot.

3. Calculating $\delta f(\varepsilon)$

As shown in the main text (Figure 1g) the photoluminescence is a first order process. To calculate $\delta f(\varepsilon)$ we take equation 2 of [13], the Boltzmann equation with electron-electron and electron-phonon scattering parameterized entirely from DFT calculations (noting electron-electron scattering includes what is sometimes referred to as Auger effects [14]) and add in a continuous wave heating term:

$$\frac{d}{dt}f(\varepsilon,t) = \Gamma_{\text{e-e}}[f](\varepsilon) + \Gamma_{\text{e-ph}}[f,T_l](\varepsilon) + \frac{p_{\text{abs}}P(\omega_{\text{in}},\varepsilon)}{g(\varepsilon)},$$

where

$$\Gamma_{\text{e-e}}[f](\varepsilon) = \frac{2D_{\text{e}}}{\hbar}\int \frac{d\varepsilon_1 d\varepsilon_2 d\varepsilon_3\big(g(\varepsilon_1)g(\varepsilon_2)g(\varepsilon_3)\big)}{g^3(\varepsilon_{\text{F}})} \times \delta(\varepsilon+\varepsilon_1-\varepsilon_2-\varepsilon_3)\{f(\varepsilon_2)f(\varepsilon_3)\big(1-f(\varepsilon)\big)\big(1-f(\varepsilon_1)\big) - f(\varepsilon)f(\varepsilon_1)\big(1-f(\varepsilon_2)\big)\big(1-f(\varepsilon_3)\big)\},$$

with D_{e} a constant of proportionality extracted from ab-initio calculations of electron lifetimes [7],

$$\Gamma_{\text{e-ph}}[f,T_l](\varepsilon) = \frac{1}{g(\varepsilon)}\frac{\partial}{\partial\varepsilon}\left[H(\varepsilon)\left(f(\varepsilon)\big(1-f(\varepsilon)\big) + k_{\text{B}}T_{\text{l}}\frac{\partial f}{\partial\varepsilon}\right)\right],$$

where $H(\varepsilon)$ is an energy-resolved electron-phonon coupling strength calculated from ab-initio electron-phonon matrix elements [7], $p_{\text{abs}}$is the absorbed power density, $P(\omega_{\text{in}},\varepsilon)$ is carrier distribution excited by a single photon of energy $\hbar\omega_{\text{in}}$ (as defined in Brown et al. [7]), $g(\varepsilon)$ is the electronic density of states (to convert from carrier number to occupation change), k_{B} is the Boltzmann constant and T_{l} the lattice temperature.

We set $\frac{df}{dt} = 0$ and perform an expansion in $f(\varepsilon)$ to linearise the right hand side. The solution to the linearised version of this equation gives the quantity $\frac{\delta f(\varepsilon)}{p_{\text{abs}}}$, which as a last step we multiply by $\hbar\omega_{\text{in}}$ to give a quantity per absorbed photon in subsequent analyses. We note this approach is equivalent to Sivan et al.'s calculation in the case of weak carrier excitation, but here interband transition elements are also included in our calculations [15]. We present $\frac{\delta f(\varepsilon)}{p_{\text{abs}}}$ in Figure S10.

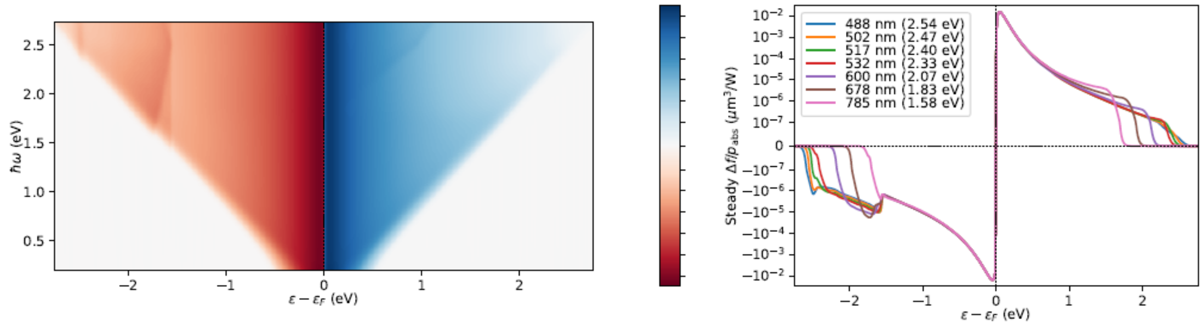

Figure S10. Steady state electron/hole population per absorbed photon as a function of laser excitation energy (ħω) and excited electron/hole energy ($\varepsilon - \varepsilon_F$).

4. Confirming black body emission theory

Here we demonstrate that our equations reduce to black body emission theory for spontaneous emission in the case of no excitation for direct transitions (i.e. confirm thermodynamic equilibrium). In equation S7 for emission of light from a point in the metal, we note that $\epsilon_{\vec{k}n'} = \epsilon_{\vec{k}n} + \hbar\omega$ and that, at equilibrium

$$f_{\vec{k}n'}\left(1 - f_{\vec{k}n}\right) = \frac{f_{\vec{k}n} - f_{\vec{k}n'}}{e^{\hbar\omega_{\text{out}}\beta} - 1}$$

where $\beta = \frac{1}{k_B T}$ and T temperature. Applying this transformation we can write the emission per unit volume, per unit energy, per unit time in the material at equilibrium as

$$\Gamma_{\text{equib}}(E) = \frac{\text{Re}\left\{\sqrt{\epsilon_{\text{m}}(\omega_{\text{out}})}\right\}\omega_{\text{out}}}{\hbar\pi^2 c^3} \times \frac{1}{e^{\hbar\omega_{\text{out}}\beta} - 1} \times \text{Im}\left(\epsilon(\omega_{\text{out}})\right) = \frac{8\pi\alpha_{\text{abs}}\text{Re}\left\{\sqrt{\epsilon_{\text{m}}(\omega_{\text{out}})}\right\}^2 E^2}{h^3 c^2 (e^{E\beta} - 1)}.$$

The emission per unit volume at equilibrium is also given by the Shockley-Van-Roosebroek formula as $4\pi\alpha_{\text{abs}}(E)\text{Re}\left\{\sqrt{\epsilon_{\text{m}}(\omega_{\text{out}})}\right\}^2 \phi_{\text{bb}}(E)$ (the definition of internal luminescence in semiconductors), which is identical to what we obtain above [16]. Here $\alpha_{\text{abs}}(E)$ is the absorption coefficient of the gold

(per unit length) and ϕ_{bb} is the black body emission flux per unit energy, per unit area, per unit solid angle.

5. Simulation results explored in more detail

There is a significant difference between $\mathrm{Re}\left\{\sqrt{\epsilon_{\mathrm{m}}(\omega)}\right\}$ from experiment, $\mathrm{Re}\left\{\sqrt{\epsilon_{\mathrm{m}}(\omega)}\right\}_{\mathrm{exp}}$, and theory, $\mathrm{Re}\left\{\sqrt{\epsilon_{\mathrm{m}}(\omega)}\right\}_{\mathrm{th}}$, especially at wavelengths longer than 600 nm (see Figure S11a). Therefore, when multiplying our DFT calculations by $\mathrm{Re}\left\{\sqrt{\epsilon_{\mathrm{m}}(\omega)}\right\}$ we used the experimental value for the plot in the main text. However, as noted in the previous section, for black-body equilibrium equation S7 reduces to $4\pi\alpha_{\mathrm{abs}}(E)\mathrm{Re}\left\{\sqrt{\epsilon_{\mathrm{m}}(\omega)}\right\}^2\phi_{\mathrm{bb}}(E)$. Therefore, it can be argued that there is still a discrepancy from the second $\mathrm{Re}\left\{\sqrt{\epsilon_{\mathrm{m}}(\omega)}\right\}$ factor. In Figure S11b we present the equivalent of Figure 3d in the main text but multiplying the luminescence by $\frac{\mathrm{Re}\left\{\sqrt{\epsilon_{\mathrm{m}}(\omega)}\right\}_{\mathrm{exp}}}{\mathrm{Re}\left\{\sqrt{\epsilon_{\mathrm{m}}(\omega)}\right\}_{\mathrm{th}}}$. It can be seen that there is better experiment-theory agreement, especially at longer wavelengths. Therefore, a possible cause of discrepancies at longer wavelengths due to the difference between the experimental and calculated values of $\mathrm{Re}\left\{\sqrt{\epsilon_{\mathrm{m}}(\omega)}\right\}$.

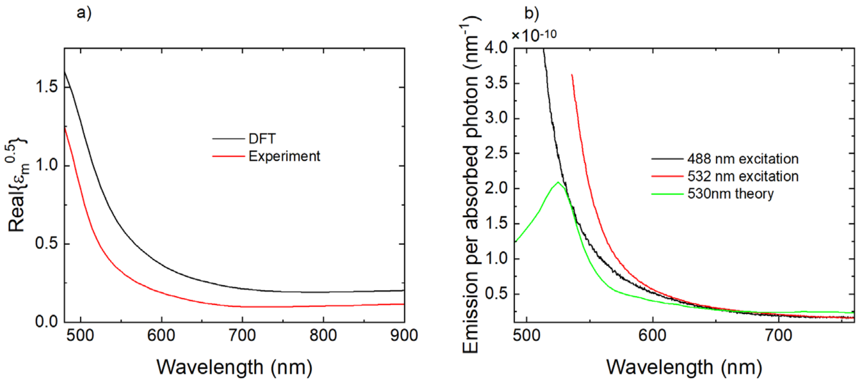

Figure S11. a) DFT and experimental values of n' as a function of wavelength. b) DFT prediction when multiplied by a factor of $\frac{\mathrm{Re}\left\{\sqrt{\epsilon_{\mathrm{m}}(\omega)}\right\}_{\mathrm{exp}}}{\mathrm{Re}\left\{\sqrt{\epsilon_{\mathrm{m}}(\omega)}\right\}_{\mathrm{th}}}$*, alongside experimental results presented in Figure 3c.*

In Figure S12a we present the simulated internal luminescence for different excitation wavelengths. In all cases the luminescence at wavelengths shorter than the excitation wavelength is due to broadening from the short lifetime of the hole in its initial excited state (see Supplemental Note 8 section 2 for more detail). For excitation at 490 nm a much stronger luminescence signal is predicted at shorter wavelengths than is observed experimentally. This is due to the simulation including a high energy d-band that is not present in the experiment (see Figure S10 or additional high energy state occupation). We note this discrepancy is well within the tolerance for a DFT calculation. The luminescence signal

can be broken down into two parts – pre- and post-scattered luminescence, as is presented in Figure S12b for 530 nm excitation.

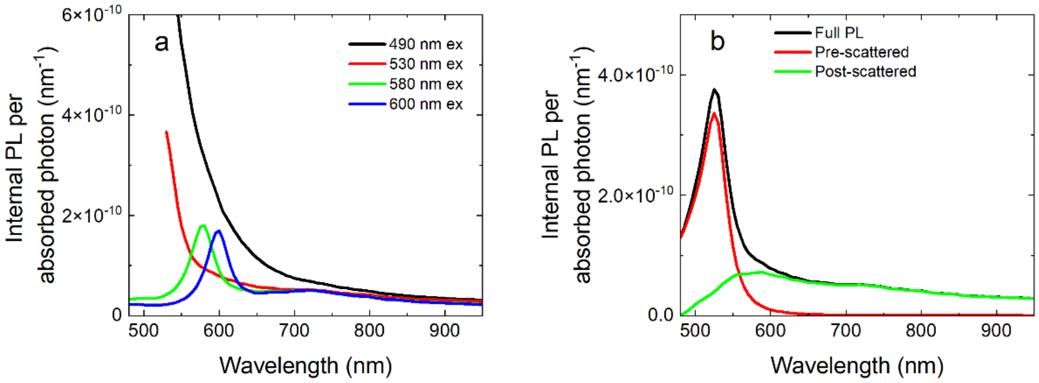

Figure S12. a) Predicted internal luminescence for different excitation wavelengths. b) The breakdown of internal luminescence into pre-scattered and post-scattered luminescence for 530 nm excitation.

In Figure S13 we present the total DFT simulated PL in energy space. We note that to generate this figure we used $\mathrm{Re}\left\{\sqrt{\epsilon_{\mathrm{m}}(\omega)}\right\}_{\mathrm{th}}$ instead of $\mathrm{Re}\left\{\sqrt{\epsilon_{\mathrm{m}}(\omega)}\right\}_{\mathrm{exp}}$ as it is parametrized much further into the infra-red region.

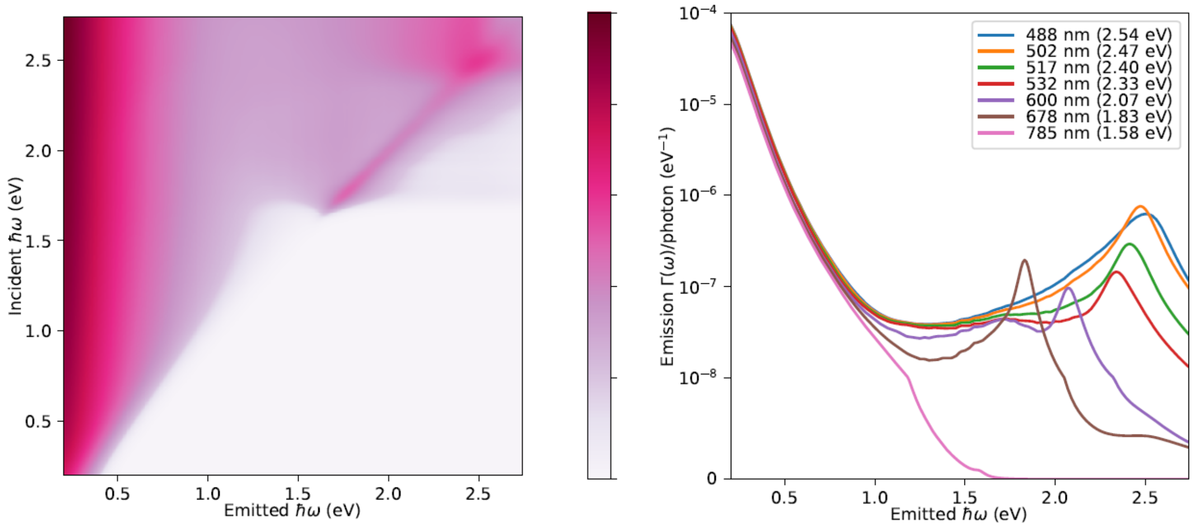

Figure S13. Steady state photoluminescence as a laser (incident) excitation energy (ħω) and emitted luminescence energy. Plot on right hand side presents lineslices for specific excitation wavelengths.

Supplemental Note 9 – lattice temperature effects

Figure 1f presents a change in luminescence with lattice temperature. Here we discuss this effect in more detail. We start by considering changes to the real part of the refractive index and absorption coefficient (proportional to the imaginary part) with temperature (Figure S14), which was measured experimentally [17].

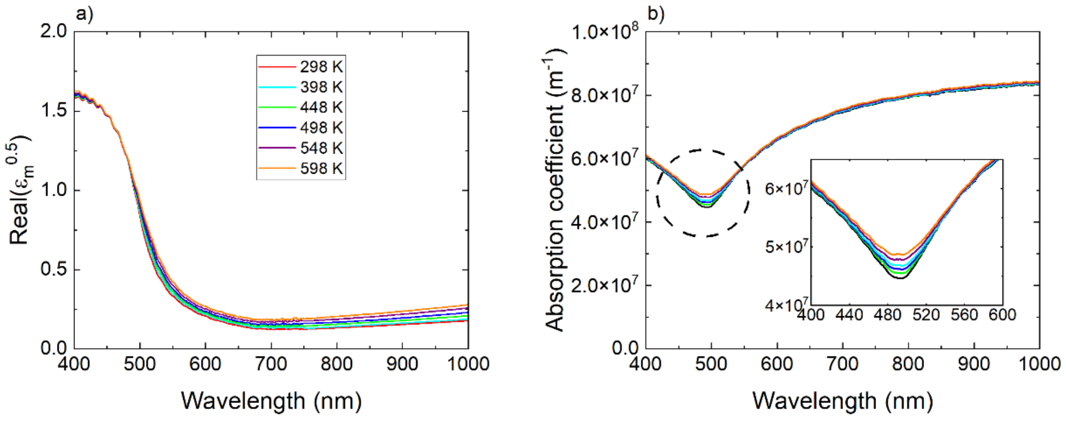

Figure S14. a) Real part of refractive index with temperature. b) Absorption coefficient with temperature. Legend in a) applies to both plots. Inset in b) expands circular dashed region.

It can be seen that while the real part of the refractive index changes at all wavelengths with temperature, the absorption coefficient mainly changes only around 500 nm. Furthermore, as at this wavelength absorption is dominated by interband transitions we suggest that this change in the absorption coefficient is primarily related to small changes in lattice constant.

We used the data presented in Figure S14 to predict how $\mathrm{Re}\{\sqrt{\epsilon_\mathrm{m}(\omega)}\}\int_0^d dz_0\, f_\mathrm{abs}(z_0,\lambda_\mathrm{in})\, f_\mathrm{emit}(z_0,\lambda_\mathrm{out})$ changes with temperature. This function contains all the factors that are explicitly a function of the refractive index and absorption coefficient. We present the function, normalised to the maximum value at 298 K, in Figure S15.

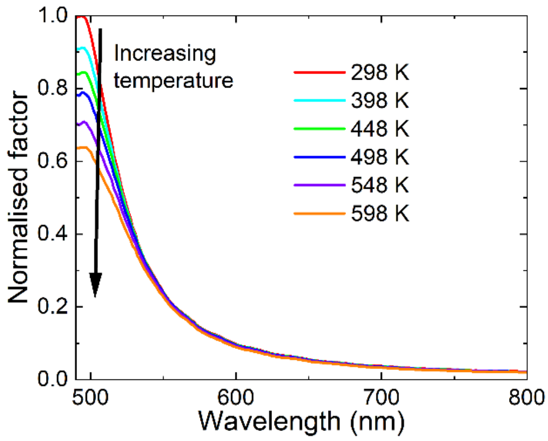

Figure S15. Change in factors that explicitly depend on the permittivity with temperature.

As can be seen in Figure S15, this factor changes in exactly the same fashion as that seen in Figure 1f, explaining that a significant temperature dependence is due to excitation and re-absorption changes, rather than a change in the fundamental luminescence properties itself. We note that the changes presented in Figure S15 do not perfectly agree in magnitude with those presented in Figure 1f i.e. it is possible that there is also some change in the internal luminescence with temperature, but that is a smaller secondary effect beyond the scope of this work. Lastly, we note that the change in the absorption coefficient that causes this effect is relatively small (Figure S14b), so even small experimental errors can have a significant impact of the magnitude of the effect seen in Figure S15.

Supplemental Note 10 – Recording the same signal when exciting and observing the signal from the glass side or air side

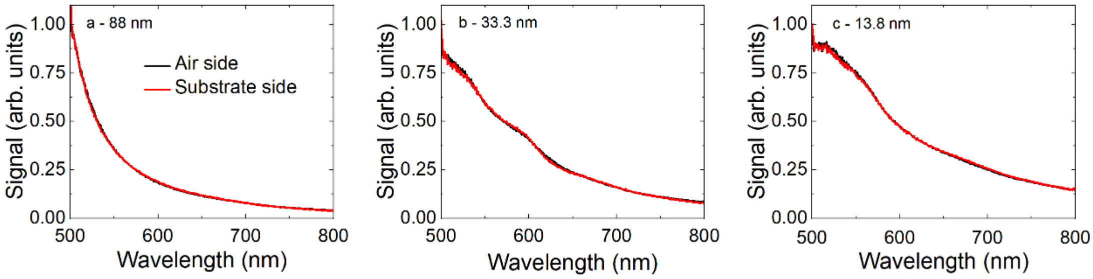

Figure S16. Photoluminescence signal when sample is excited and signal is observed from the air side (black curve), or through the substrate (red curve), with 488 nm excitation, and for different sample thicknesses as indicated on the inset. Incident intensity was 0.017 mWμm^{-2} *when exciting through substrate and 0.079 mWμm*$^{-2}$ *when exciting from air side.*

Supplemental Note 11 – Further discussion of thin flake photoluminescence

The model presented in the main text originates as follows: the wavevector in the direction perpendicular to the interface becomes discretised with $\frac{2\pi}{d}$ spacing in k-space. This gives a corresponding energy of $\hbar v_f n \times \frac{2\pi}{d} = \frac{hnv_f}{2d}$, where v_f is the fermi velocity and n is in integer spacing.

We note that there are other ways to fit the data presented in Figure 4c. We present a different fit via an experimentally motivated approach in Figure S17. Specifically, all energy shifts via a single equation: $Energy\ shift = ntK$, where n is an integer (in the plot we observe n =1, 2, 4 and 6) and K a fitting parameter which we record as (6.8±0.1) meV nm^{-1}. In the Figure S17 inset we plot energy shifts divided by n: it can be see that all resonance peaks lie on one straight line.

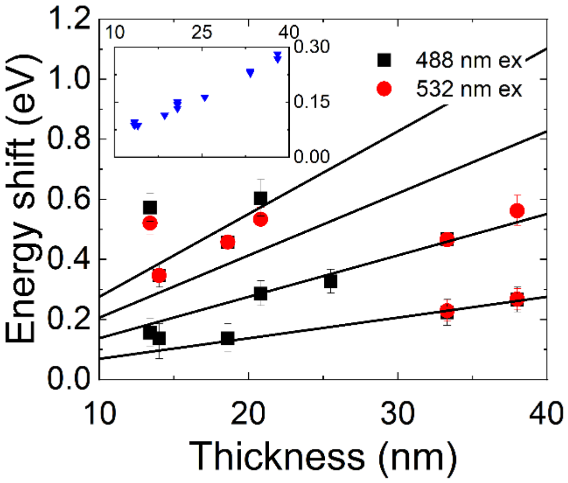

Figure S17. Alternative linear fitting of the data presented in Figure 4c (see text for details).

Supplemental Note 12 – DFT calculations of the thickness-dependent electronic structure

In contrast to coherent light scattering associated with, for example, linear polarization diagrams and nonlinear harmonic generation, PL is a form of inelastic light scattering in the sense that incident photons are converted into PL photons emitted with different energy and without maintaining temporal coherence relative to the incident light. The energy difference in PL is absorbed by excitations in the material such as electronic and phononic modes. Incident light commonly triggers electronic transitions. Excited charge carriers involved in these transitions interact with other electrons and/or ions in the material, evolving in a complex cascade dynamic consisting of the creation of multiple secondary excitations that engage other charge carriers (i.e., holes and electrons) as well as vibrational modes. These processes can delay the radiative emission part of the inelastic scattering process relative to the absorption part, depending on the lifetimes of the intermediate electronic and/or phononic states. One can then classify PL emission contributions into two broad categories: (i) fast radiative transitions taking place when charge carriers that are directly excited by the external light undergo a transition to another lower-energy state in a different band (i.e., interband radiative emission). The transition can further involve the creation of atomic vibrations in a Raman-like fashion; (ii) slow emission taking place after the excited charge carriers have undergone some intra- or interband dynamics, and eventually decay radiatively to lower energy states.

As we discussed in the first part of the manuscript, photoluminescence from pre-scattered carriers is particularly sensitive to the details of the available electronic states. We propose that the most likely cause of resonance effects is related to the quantisation of bands near the Fermi level for thin samples, coupled to pre-scattered luminescence. In Figure S18 we present a schematic that compares the process presented in Figure 3e of the main text with the situation we propose here for thin flakes.

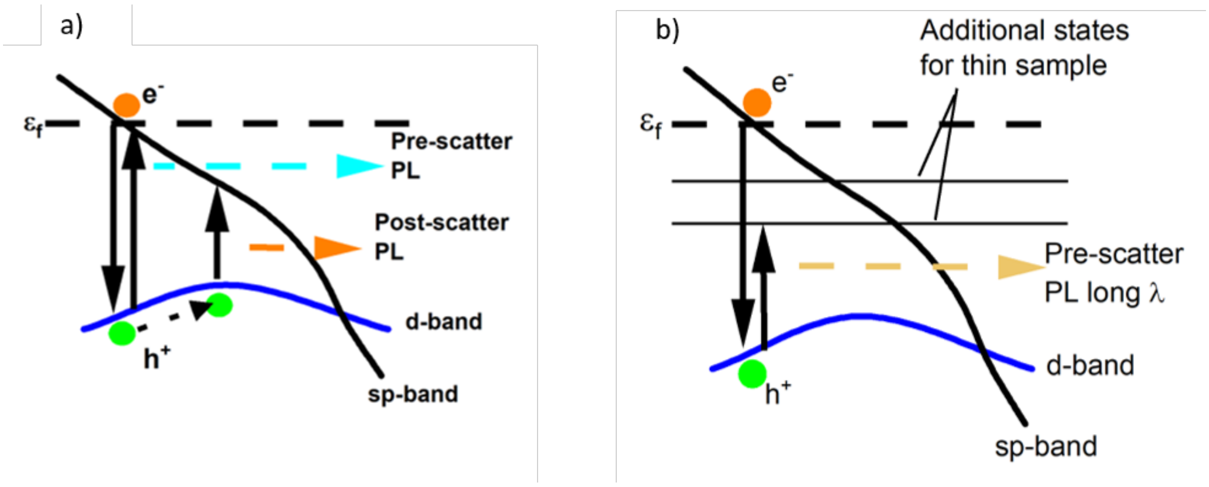

Figure S18. Schematics comparing luminescence processes in thick flakes, a), and thin flakes, b). For thin flakes pre-scattered luminescence can occur at longer wavelengths due to quantization of bands near the Fermi level.

To further study band-structure effects on the observed photoluminescence, we performed first-principles calculations based on density-functional theory (DFT) on films with different thicknesses up to 40 (111) atomic planes. Calculation details are given in a recent study [18]. After calculating the Kohn-Sham electronic structure and eigenvalues using the Quantum Espresso package [19], we obtain transition dipole matrix elements using the YAMBO program [20] at the 21 nearest k-points around the M point of the Brillouin zone. Subsequently, we calculate the square of dipole matrix elements, $d^2_{\varepsilon,\varepsilon'} = |\langle\varepsilon|x|\varepsilon'\rangle|^2 + |\langle\varepsilon|y|\varepsilon'\rangle|^2$, as a function of initial and final energy states and averaged over k-points in the vicinity of the M point using the following equation

$$|\langle\varepsilon|x|\varepsilon'\rangle|^2 = \frac{\sum_{n,m,\boldsymbol{k}} \delta(\varepsilon' - \varepsilon_{m\boldsymbol{k}})\delta(\varepsilon - \varepsilon_{n\boldsymbol{k}})|\langle n\boldsymbol{k}|x|m\boldsymbol{k}\rangle|^2}{\sum_{n,m,\boldsymbol{k}} \delta(\varepsilon_i - \varepsilon_{m\boldsymbol{k}})\delta\left(\varepsilon_f - \varepsilon_{n\boldsymbol{k}}\right)},$$

where delta functions are replaced by a normalized Gaussian of width σ = 50 meV. We plot $d^2_{\varepsilon,\varepsilon'}$ for different layer thicknesses in Figure S19.

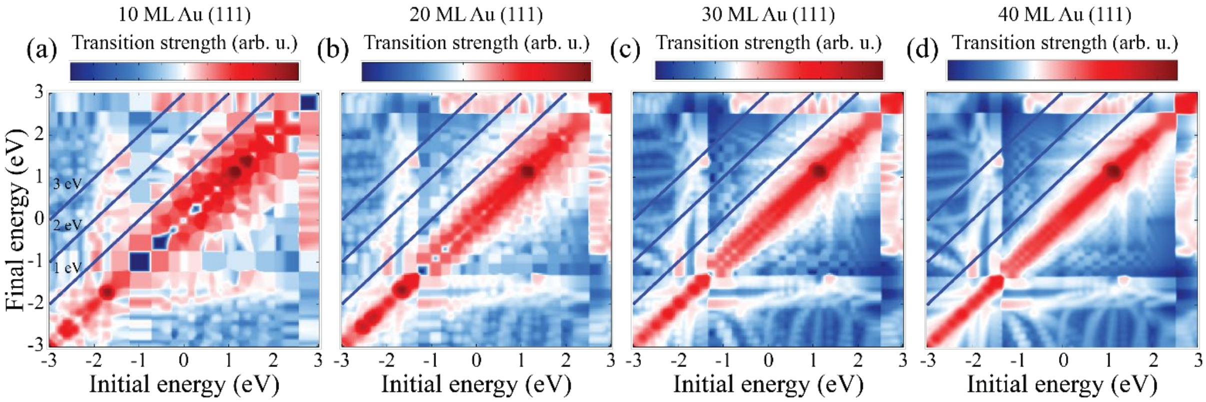

Figure S19. Averaged dipole matrix elements of Au (111) films consisting of a) 10 atomic layers, b) 20 atomic layers, c) 30 atomic layers, and d) 40 atomic layers. Blue lines are drawn to indicate transition energies between initial and final states corresponding to 1, 2 and 3 eV. Blue and red colours in the colour bar illustrate the minimum and maximum transition strengths, respectively.

Supplemental Note 13 – Results under excitation with a laser of 785 nm wavelength

Figure 1a in the main text shows that it was possible to measure luminescence from gold flakes following 785 nm excitation. We presented the signal per absorbed photon, where the absorption was based on the simulated absorption value (due to a larger error in the experimental value). We also note that for thinner flakes sample absorption is much stronger (and thus carries less error) in this wavelength region.

Here we discuss this measurement and its interpretation in detail. We begin by noting that this signal was extremely challenging to measure – at this wavelength gold absorbs less than 5 % for all thicknesses, and the luminescence efficiency is less than 10^{-10} at all wavelengths. This meant that even small noise from the instrument competed with the observed signals. We present recorded luminescence signals in Figure S20a for four flake thicknesses. The luminescence increases for thinner flakes, in agreement with our model. We found that a small portion of the observed signal originated from noise from within the microscope rather than the sample, with peaks at 870 nm and 900 nm (marked 'instrument' on the figure). Despite exploring different microscope configurations, we were unable to fully remove these signals. These peaks had a different spatial spread to the gold luminescence and were also observed when measuring a silver mirror, confirming they did not originate from the gold. To first approximation we can describe the signal as $Signal = Signal_{\text{gold}} + Signal_{\text{instrument}}$, with the second term approximately constant as flake thickness changes. To deconvolute these two components we subtracted the signal from two flakes of different thicknesses i.e. $Signal(33.3nm) - Signal(88nm) = Signal_{\text{gold}}(33.3nm) - Signal_{\text{gold}}(88nm)$. From this point we proceeded with the same analysis as presented in the main text.

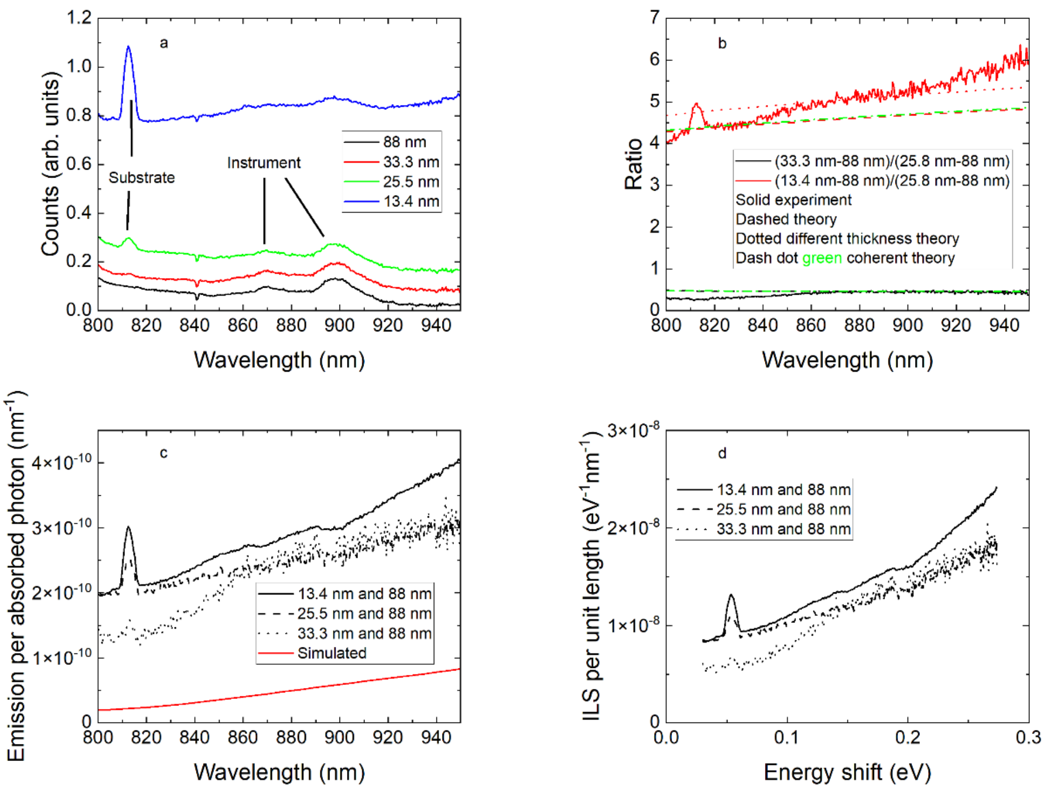

Figure S20. a) Signal counts per incident power following 785 nm excitation at 1.87 mWμm^{-2}. Substrate Raman peaks and additional signals from the instrument (see text) are marked. b) Ratios between signals presented in a), as described in the legend, alongside dashed lines based on our model prediction, and dotted lines assuming the thinnest flake is 1 nm thinner and other flake thicknesses unchanged. The theory for a fully coherent process is overlaid in green. c) the internal photoluminescence per absorbed photon, based on three different flake combinations (see figure). The simulated signal based on photoluminescence is in red. d) the strength of Inelastic Light Scattering (ILS) per unit length, as extracted from the three different flake combinations.

We present the ratio of recorded signals in Figure S20b, specifically of the type $\frac{Signal(33nm)-Signal(88nm)}{Signal(25.8nm)-Signal(88nm)}$. Overlaid on this plot in dashed lines is the equivalent to equation 2 in the main text for the different thicknesses. Here we normalise the signals by that from 25.8 nm as this flake gives a relatively strong signal and has less uncertainty in thickness than the 13.4 nm flake. However, even a small difference in the thickness can result in a significantly different predicted ratio: overlaid on this plot is a ratio setting the thinnest flake thickness to 12.5 nm (i.e., approximately 1 nm difference). This reveals that, within the experimental error of measured thicknesses (±2 nm), we find experiment and theory are in relatively good agreement. Importantly, unlike for the case of exciting at 488 nm or 532 nm, our theory agrees well with experiment for all thicknesses and we see no additional resonance features at longer wavelengths for thin flakes. We also overlay the predictions from theory assuming a fully coherent process – it can be seen that the results are almost identical. Figure S20b also

demonstrates that our approach is relatively successful in removing the instrument contribution to the signal.

We used the same approach as in the main text to calculate the internal PL per absorbed photon, which we present in Figure S20c for three different flake combinations. In all cases we obtain a very similar shape to the internal PL. Overlaid on this plot in red is the internal PL predicted from our DFT-parameterized simulations. The simulated PL is approximately a factor of 5 weaker than the experimental signal.

We further confirm that the internal PL should be on the order of 10^{-11} without reference to DFT via a simple order of magnitude calculation. To first order, gold's absorption coefficient is the same strength at 488 nm and 785 nm (Figure S21), implying that the total transition rate is the same for the two cases. For 488 nm excitation, photon absorption only populates a small region in the energy range of the d-band. In contrast, when exciting at 785 nm all energies from 1.58 eV below the fermi level (785 nm in energy) to the fermi level are approximately equally populated (as this proceeds via phonon-assisted transitions). Finally, the scattering rate is approximately an order of magnitude lower for sp-band holes, meaning they have a 'lifetime' in their current state approximately 10 times longer. Assuming equal optical transition rates between all energies, we can estimate the probability of luminescence within 0.1 eV of the excitation energy as being $2 \times 10 \times \left(\frac{0.1}{2\times1.58}\right)^2$ less probable for 785 nm excitation than 488 nm excitation. Here the factor of 2 comes from both electrons and holes contributing to the photoluminescence equally for 785 nm excitation, the factor of 10 from the lifetime and the squared factor from considering: i) the proportion of the transition strength for absorption which populates high energy holes; ii) the proportion of the emission transition strength which we can apportion to these high energy holes. This 'back of the envelope' shows, without DFT calculations, that we expect a photoluminescence strength close to the excitation wavelength to be approximately an order of magnitude weaker following 785 nm excitation than for 488 nm and 532 nm excitation (as predicted in our DFT simulations on the plot).

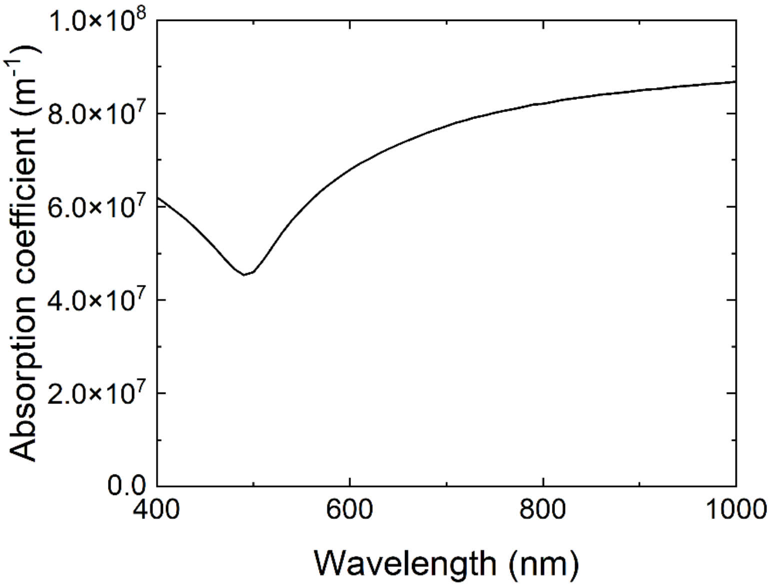

Figure S21. Absorption coefficient of gold as a function of wavelength.

Our data shows that the luminescence signal observed following 785 nm excitation is a factor of 5 larger than is reasonable if we assume its origin is luminescence. Therefore, we suggest that exciting in the intraband regime produces other Inelastic Light Scattering (ILS) type processes beyond PL. The equivalent to the model presented in the main text for ILS is

$$ILS_{\text{external}}(\lambda_{\text{out}}) = ILS_{\text{internal}}(\lambda_{\text{em}}, \lambda_{\text{out}}) \left(\frac{I_{\text{in}}(\lambda_{\text{in}})}{\hbar\omega_0}\right) \int \frac{f_{\text{abs}}(z, \lambda_{\text{in}}) f_{\text{emit}}(z, \lambda_{\text{out}})}{\alpha_0} dz$$

where α_0 is the absorption coefficient at the incident laser energy, and ILS_{internal} is the probability of internal inelastic light scattering both per unit wavelength and per unit length within gold (this includes both PL and non-PL contributions). The main change compared to the main text is to state inelastic light scattering is per laser intensity locally, rather than per absorbed photon locally. It is more typical to present ILS effects as an energy shift, so in Figure S20d we present ILS_{internal} as a function of energy shift for the three different flake combinations considered. We note this factor includes both ILS and PL contributions.

References

1. García de Abajo, F. J. *Colloquium*: light scattering by particle and hole arrays. *Reviews of Modern Physics* **79**, 1267-1290 (2007).
2. Kiani, F. & Tagliabue, G. High aspect ratio au microflakes via gap-assisted synthesis. *Chemistry of Materials* **34**, 1278-1288 (2022).
3. PHASIS. Epitaxial gold Au(111) thin film grown on mica substrate. at https://www.phasis.ch/products/epitaxial-gold-au-111 URL. [accessed on 22/01/2024]
4. Blanco, L. A. & García de Abajo, F. J. Spontaneous light emission in complex nanostructures. *Physical Review B* **69**, 205414 (2004).
5. L. Novotny and B. Hecht, *Principles of Nano-Optics*, 2nd ed. (Cambridge University Press, The Edinburgh Building, Cambridge, CB2 8RU, 2012).
6. J. D. Jackson, *Classical Electrodynamics* (John Wiley & Sons, U.S.A., 1999).
7. Brown, A. M. et al. *Ab initio* phonon coupling and optical response of hot electrons in plasmonic metals. *Physical Review B* **94**, 075120 (2016).
8. P. Würfel, *The Chemical Potential of Luminescent Radiation*, J. Phys. C: Solid State Phys. **15**, 3967 (1982).
9. McPeak, K. M. et al. Plasmonic films can easily be better: rules and recipes. *ACS Photonics* **2**, 326-333 (2015).
10. Großmann, S. et al. Nonclassical optical properties of mesoscopic gold. *Physical Review Letters* **122**, 246802 (2019).
11. Sundararaman, R. et al. JDFTx: software for joint density-functional theory. *SoftwareX* **6**, 278-284 (2017).
12. A. Drezet. Description of Spontaneous Photon Emission and Local Density of States in the Presence of a Lossy Polaritonic Inhomogeneous Medium. *Physical Review A* **95**, 043844 (2017W).
13. Brown, A. M. et al. Experimental and *ab initio* ultrafast carrier dynamics in plasmonic nanoparticles. *Physical Review Letters* **118**, 087401 (2017).
14. Lee, S. A. et al. *D*-Band hole dynamics in gold nanoparticles measured with time-resolved emission upconversion microscopy. *Nano Letters* **23**, 3501-3506 (2023).
15. Sivan, Y. & Dubi, Y. Theory of "hot" photoluminescence from Drude metals. *ACS Nano* **15**, 8724-8732 (2021).
16. van Roosbroeck, W. & Shockley, W. Photon-radiative recombination of electrons and holes in germanium. *Physical Review* **94**, 1558-1560 (1954).
17. Magnozzi, M. et al. Plasmonics of Au nanoparticles in a hot thermodynamic bath. *Nanoscale* **11**, 1140-1146 (2019).
18. Rodríguez Echarri, A. et al. Nonlinear photoluminescence in gold thin films. *ACS Photonics* **10**, 2918-2929 (2023).
19. Giannozzi, P. et al. QUANTUM ESPRESSO: a modular and open-source software project for quantum simulations of materials. *Journal of Physics: Condensed Matter* **21**, 395502 (2009).
20. Marini, A. et al. Yambo: an *ab initio* tool for excited state calculations. *Computer Physics Communications* **180**, 1392-1403 (2009).
